# Comparative genomic analyses on assassin bug *Rhynocoris fuscipes* (Hemiptera: Reduviidae) reveal genetic bases governing the diet-shift

**DOI:** 10.1016/j.isci.2024.110411

**Published:** 2024-06-28

**Authors:** Ling Ma, Yuange Duan, Yunfei Wu, Hailin Yang, Haibin Deng, Xinzhi Liu, Tianyou Zhao, Yisheng Zhao, Li Tian, Fan Song, Teiji Sota, Wanzhi Cai, Hu Li

**Affiliations:** 1 Department of Entomology and MOA Key Lab of Pest Monitoring and Green Management, College of Plant Protection, China Agricultural University, Beijing 100193, China; 2 College of Biology and Food Engineering, Chuzhou University, Chuzhou 239000, China; 3 Yunnan Tobacco Company, Yuxi Branch, Yuxi 653100, China; 4 Tobacco Research Institute of Guangdong Province, Shaoguan 512029, China; 5 Department of Zoology, Graduate School of Science, Kyoto University, Kyoto, Sakyo 606-8502, Japan

**Keywords:** Entomology, Nucleic acids, Genomics, Epigenetics, Phylogenetics, Biological classification, Transcriptomics

## Abstract

Genetic basis underlying the biodiversity and phenotypic plasticity are fascinating questions in evolutionary biology. Such molecular diversity can be achieved at multi-omics levels. Here, we sequenced the first chromosome-level genome of assassin bug *Rhynocoris fuscipes*, a polyphagous generalist predator for biological control of agroecosystems. Compared to non-predatory true bugs *Apolygus lucorum* and *Riptortus pedestris*, the *R. fuscipes*-specific genes were enriched in diet-related genes (e.g., serine proteinase, cytochrome P450) which had higher expression level and more exons than non-diet genes. Extensive A-to-I RNA editing was identified in all three species and showed enrichment in genes associated with diet in *R. fuscipes*, diversifying the transcriptome. An extended analysis between five predaceous and 27 phytophagous hemipteran species revealed an expansion of diet-related genes in *R. fuscipes*. Our findings bridge the gap between genotype and phenotype, and also advance our understanding on genetic and epigenetic bases governing the diet shifts in ture bugs.

## Highlights

• Chromosome-level genome of assassin bug *Rhynocoris fuscipes* was assembled
• Diet-related genes were overrepresented and highly expressed in *R. fuscipes*
• Extensive A-to-I RNA editing was enriched in diet genes, increasing gene diversity

## Graphical abstract

**Figure:**
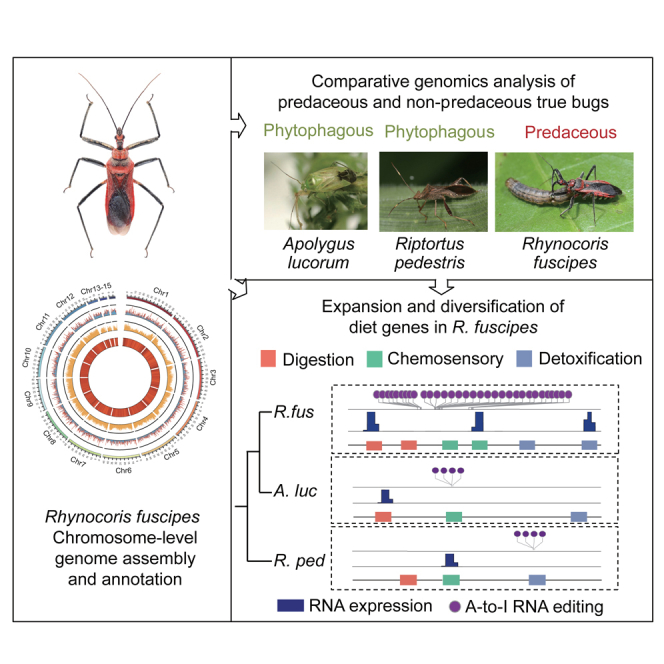

## Introduction

Biodiversity of the tree of life and the phenotypic innovations of organisms during evolution have long been the most fascinating questions to evolutionary biologists. Clarifying the genetic basis and molecular mechanisms governing the phenotypic diversity would bridge the gap between genotype and phenotype, help us better understand different layers of biodiversity, and might contribute to the preservation of biological resources.

Insects are the most diversified clade in the animal kingdom.^1^^,^^2^^,^^3^ One of the most interesting phenotypic diversities in insects is the diet-shift. Insects exhibit a wide variety of feeding strategies like predaceous, phytophagous, hematophagous, and omnivorous. These traits might have independently emerged in different insect clades responding to selection pressure and environmental adaptation. Predaceous bugs, in particular, have great ecological and economic value. For example, integrated pest management used predatory arthropods as biocontrol agents and resulted in enormous benefits in agriculture,^4^^,^^5^^,^^6^ highlighting the necessity of studying diet traits. However, the molecular mechanisms underlying diet difference is poorly understood in a larger evolutionary scale. A recent study on true bugs proposed that diet-related genes in predaceous species might be acquired by horizontal gene transfer, but how these genes are selectively maintained and how they function and cooperate with the existing molecular network remain to be investigated.

Among the various predaceous bugs, some are specialist predators such as millipede assassins^7^ and termite assassins,^8^ and some are generalist predators.^9^ The insect family Reduviidae (Hemiptera: Heteroptera: Cimicomorpha) contains ∼7,500 species belonging to 990 genera and more than 20 subfamilies.^10^^,^^11^ Most of them are generalist predators^12^ except for a few blood-sucking species of the subfamily Triatominae.^13^ Reduviidae species are of special interest due to the following reasons: (1) they generally have larger body sizes than many other predaceous hemipterans, providing convenience in mass-rearing, releasing, and management of the bugs; (2) Reduviidae predators feed on a much wider range of preys compared to other predaceous bugs,^14^^,^^15^ making them suitable for controlling different kinds of pests in the fields.^16^^,^^17^ Therefore, compared to the various non-predaceous insects, Reduviidae predators represent a successful diet-shift in insects that benefit humans in many ways. However, apart from phenotypic characterization of these Reduviidae species, the genetic determinants of their predatory behavior remain elusive. There is an urgent need in understanding the molecular foundation and evolutionary dynamics of diet-shift in Reduviidae or even the insect clade.

**Figure 1:**
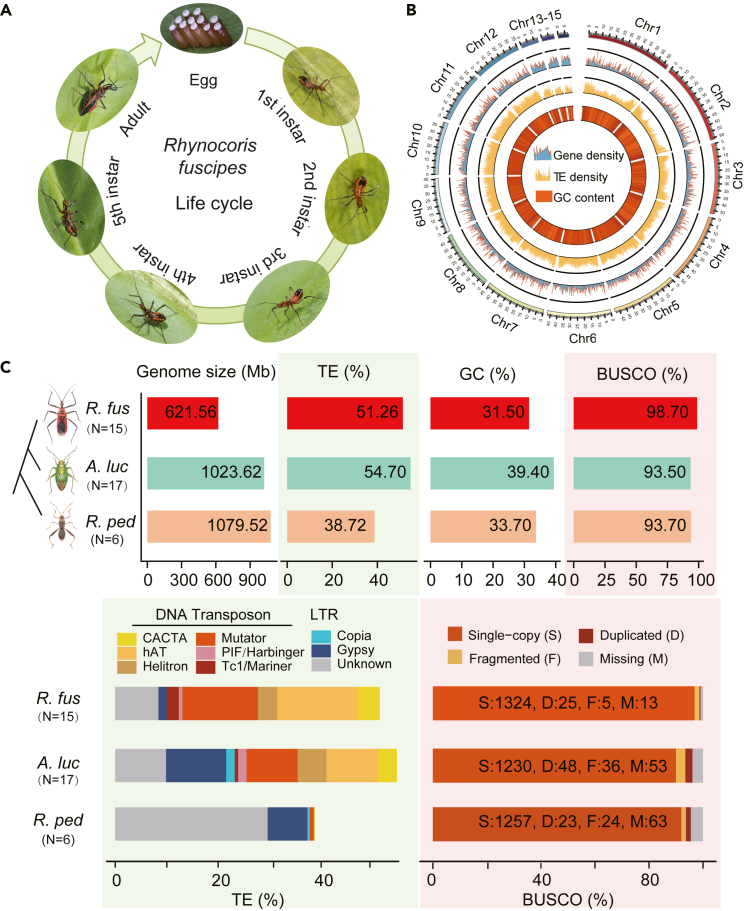
Genome assembly and characterization of *Rhynocoris fuscipes* (A) Images of *R. fuscipes* highlighting its predatory behavior. Different developmental stages were displayed. (B) Circus plot of the *R. fuscipes* genome. The different layers of circle indicate the distribution of gene density (blue), TE density (gold), and GC density (dark orange). Densities were calculated in 100 kb bins. (C) The genomic composition of *R. fuscipes* and other two non-predaceous true bug with high genome quality (*A. lucorum* and *R. pedestris*). Species were ordered by their phylogeny. The genomic features include genome size, TE content, GC content, and BUSCO (benchmarking universal single-copy orthologs) assessment of genome completeness. The TE contents were further shown by different subfamilies of LTRs (long terminal repeats) and DNA transposons. The BUSCO results were further classified into single-copy (S), duplicated (D), fragmented (F), and missing (M) genes.

Assassin bug *Rhynocoris fuscipes* (denoted as *R. fuscipes*) is an important predator for crop pests such as larvae of *Helicoverpa armigera* and *Spodoptera litura* (Lepidoptera).^18^ *R. fuscipes* has five nymph periods before adult, and both immature bugs and adults are predaceous (Figure 1A), with a high level of daily prey consumption.^19^^,^^20^ It was reported that *R. fuscipes* feeds on more than 40 pest species of agricultural concern.^17^^,^^19^ Therefore, *R. fuscipes* has been massively reared as a biological control agent in regions like Southeast Asia.^17^^,^^21^ The unique wide spectrum of preys makes *R. fuscipes* as an ideal model for studying the diet-shift during insect evolution. Several studies have characterized *R. fuscipes* from different phenotypic aspects,^16^^,^^21^ but the genetic basis underlying its unique phenotype/behavior remains poorly understood. Uncovering the molecular determinants of its strong predaceous trait and phenotypic diversity/complexity would bridge the gap between genotype and phenotype, advancing our understanding on this fundamental question in life science.

According to the central dogma, genetic information flows from DNA to RNA, and then to protein.^22^ As protein is the main executor of molecular functions, the proteome largely determines the phenotype of an organism. However, proteomic complexity could be achieved at multiple layers. (1) Genomic level: the presence/absence of a DNA (gene) sequence directly determines the corresponding phenotype while DNA polymorphisms might fine tune the protein function or diversity; (2) transcriptomic level: some post-transcriptional (or epigenetic) *cis*-regulatory mechanisms like alternative splicing, RNA modifications, and temporal-spatial gene expression could also diversify the existing proteins without changing the DNA sequence. Therefore, the phenotypic innovations/uniqueness of a species could result from either the existence of a particular gene or from the post-transcriptional processing and RNA modifications.

A-to-I RNA editing, mainly mediated by ADAR (adenosine deaminase acting on RNA) proteins, is one of the best-studied RNA modifications in all kingdoms of lives.^23^^,^^24^^,^^25^ Since I is read as G, A-to-I RNA editing leads to similar consequences of A-to-G mutation. However, compared to the potential pleiotropic effects caused by DNA mutations, RNA editing can diversify the transcriptome in a temporal-spatial manner.^26^^,^^27^^,^^28^^,^^29^^,^^30^ The same advantage goes for alternative splicing. Therefore, A-to-I RNA editing and alternative splicing are regarded as two major sources to achieve flexibility under different environmental conditions or during the development of a species.^31^^,^^32^ These epigenetic regulatory mechanisms could alter the genetic information and promote adaptation beyond the DNA sequence.^33^

In this study, we sequenced and assembled a new chromosome-level genome (621.56 Mb) of assassin bug *Rhynocoris fuscipes* (*R. fuscipes*). Combined with two additional high-quality public genomes of non-predaceous true bugs *Apolygus lucorum* (*A. lucorum*) and *Riptortus pedestris* (*R. pedestris*) together with other representative Paraneoptera species, we performed comparative genomic and evolutionary analyses. We found that both genomic and transcriptomic regulations might contribute to the formation of diet trait in *R. fuscipes*, providing a highly possible explanation for how *R. fuscipes* acquired the predatory phenotype and the corresponding digestive capacity. We also propose that in this multi-omics era, the molecular mechanisms underlying the adaptive phenotypes could be understood at multiple layers that enable us draw a comprehensive regulatory network.

## Results

### Genome assembly and annotation of assassin bug *Rhynocoris fuscipes*

To understand the genetic basis governing the predatory phenotype of *R. fuscipes* (Figure 1A), we sequenced, assembled, and annotated the chromosome-level genome of this assassin bug. In total, 49.64 Gb Illumina short-reads (∼88.11 × coverage) were obtained after a preliminary filtering step (Table S1 and method details). Based on 17-mer depth, the genome size was estimated to be 563.39 Mb with 1.81% heterozygosity (Table S2; Figure S1A). Genome assembly was performed using 26.19 Gb PacBio HiFi reads (∼46.49 × coverage). This contig-level genome size was 621.03 Mb with contig N50 of 3.93 Mb and a maximum contig length of 34.08 Mb (Table S3). After physical mapping with 55.39 Gb Hi-C clean data, 597.78 Mb (96.17%) of the assembled sequences were anchored onto 15 linkage groups that were well distinguished in the genome landscape (Figure 1B; Figure S1B). This resulted in a final chromosomal-level genome of 621.56 Mb (Table 1) with scaffold N50 of 47.56 Mb, a maximum chromosome length of 63.53 Mb (Chr1), and a genome-wide GC content of 31.50% (Figure 1C; Table S3). Next, we found that 94.65% of the Illumina short reads could be mapped to the reference genome, suggesting the high-quality of our genome assembly. Accordingly, the genome completeness of *R. fuscipes* was as high as 98.70% as estimated by BUSCO (Figure 1C).

**Table 1:** Statistics for genome assembly and annotation of predaceous *Rhynocoris fuscipes* and other two non-predaceous true bugs

Item	Predaceous	Non-predaceous	
	*Rhynocoris fuscipes*	*Apolygus lucorum*^34^	*Riptortus pedestris*^35^
Assembly level	Chromosome	Chromosome	Chromosome
Assembly size (Mb)	621.56	1023.62	1079.52
Contig N50 (Mb)	3.93	0.78	12.09
Scaffold N50 (Mb)	47.56	68.13	161.37
Annotated genes	17,486	20,353	19,026
Repetitive content (%)	51.26	54.70	38.72
GC rate (%)	31.50	39.40	33.70

A total of 17,486 genes were annotated in the *R. fuscipes* genome, with an average CDS length of 1,218 bp, average mRNA length of 1573 bp, and average exon number of 7 (Table S4). Based on the synteny analysis of genes on each chromosome, we postulate that chrX is likely to be the sex chromosome of *R. fuscipes* (see STAR methods for details). We then identified that 318.64 Mb (51.26%) of the *R. fuscipes* genome belonged to transposon elements (TEs) (Table 1; Table S5), among which DNA transposons were the most abundant TE category (256.90 Mb, 41.33%). The major super-families of DNA transposons included hAT (15.71%), Mutator (14.68%), CACTA (4.19%), and Helitron (3.72%) (Figure 1C; Table S5).

Next, to make a deep and accurate comparative genomic analysis on how the predatory phenotype of *R. fuscipes* was achieved and evolved, we need the high-quality genomes of several non-predaceous insects as a control. We restricted our analysis to true bugs (Heteroptera), the largest suborder of Hemiptera. Under multiple criteria involving whether a species had a chromosome level genome with gene annotation and a matched transcriptome (see STAR methods for the detailed criteria), we finally focused on two non-predaceous true bugs *Apolygus lucorum* (*A. lucorum*) and *Riptortus pedestris* (*R. pedestris*). *A. lucorum*is mainly phytophagous (sometimes omnivorous) and *R. pedestris* is strictly phytophagous.

The genomes of these three species were generally of high quality but our *R. fuscipes* still had higher genome completeness compared to other two non-predaceous true bugs (Figure 1C). The proportions of genomic repeats varied greatly across three species. *A. lucorum* had the highest repeats content (54.70%) compared to *R. fuscipes* (51.26%) and *R. pedestris* (38.72%) (Table 1; Figure 1C). Interestingly, when we looked at different types of TEs, including LTR (long terminal repeat) and DNA transposon, we found that LTR contents were considerably lower in *R. fuscipes* than in the two non-predaceous species, while DNA transposons like Mutator and hAT were much more abundant in *R. fuscipes* compared to the other two bugs (Figure 1C). This might indicate that this predator insect *R. fuscipes* has experienced an expansion of repetitive elements in the genome (or the two non-predaceous species experienced gene contraction), mainly for the Mutator and hAT super-families of DNA transposons.

### Evolutionary genomic analyses reveal strong enrichment of diet-related functions in *R. fuscipes*-specific genes

**Figure 2:**
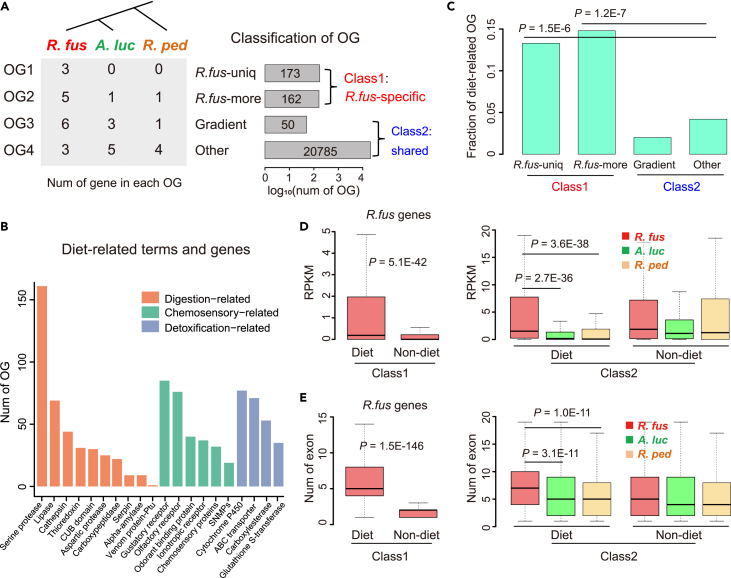
Enrichment of diet-related genes in *R. fus*-specific genes (A) Schematic diagram illustrating the definition and numbers of *R. fus*-specific (class1, red) and shared (class2, blue) ortho-groups. OG, ortho-group. (B) Collection of diet-related terms and genes. The numbers of genes in each term were shown as barplot. (C) Fraction of diet-related OGs in each category. *p* values were calculated with Fisher’s exact tests. (D) Gene expression (measured by RPKM) of each category. *p* values were calculated with Wilcoxon rank sum tests. (E) Numbers of exons per gene in each category. *p* values were calculated with Wilcoxon rank sum tests.

To bridge the gap between phenotype and genotype, we need to understand the genetic basis of how *R. fuscipes* adapts to predaceous behavior together with the corresponding diet. We first collected all currently known genes associated with diet (hereinafter called diet-related genes or diet genes) (method details). With the high-quality genomes of *R. fuscipes* and other two non-predaceous true bugs (*A. lucorum* and *R. pedestris*), our general strategy is to divide all genes into *R. fus*-specific genes (Class1) and shared genes (Class2) (Figure 2A for the most reasonable criteria; Figure S2 for other cutoffs) and then compare the enrichment of diet-related functions in these two classes. If there is particular enrichment of diet genes in *R. fus*-specific genes, then it will potentially account for the unique predaceous-related feature of *R. fuscipes*.

We totally obtained 21,170 ortho-groups (OGs) among *R. fuscipes*, *A. lucorum*, and *R. pedestris* (Table S6, also see STAR methods for the detailed bioinformatic procedures). According to the number of genes in each OG, we first defined *R. fus*-unique OG as *R. fus* > 2 & *A. luc* = 0 & *R. ped* = 0, resulting in 173 OGs (Figure 2A for the main results; Figure S2 for the robustness under other cutoffs). However, the *R. fus*-specific phenotype could not only be attributed to the *R. fus*-unique OGs but also might be caused by the OGs overrepresented in *R. fuscipes* compared to *A. lucorum* and *R. pedestris*. Therefore, we further defined the “*R. fus*-more” category and found 162 OGs (Figure 2A). Then, the 173 *R fus*-unique OGs and 162 *R. fus*-more OGs are collectively defined as “*R. fus*-specific” OGs (Class1, 335 OGs). The remaining 20,835 OGs were classified as Class2, termed “shared OGs”, including 50 “gradient” OGs with *R. fus* > 1.5×*A. luc* and *A. luc* > 1.5×*R. ped* (see STAR methods for the detailed criteria) and 20,785 “Other” OGs (Figure 2A). The reason for defining the “gradient” OGs is that *A. lucorum* is not strictly phytophagous (sometimes it is omnivorous), we therefore reserve the possibility that some genomic features of *A. lucorum* might be intermediate between *R. fuscipes* and *R. pedestris*. According to the above criteria, 98.4% (20,835/21,170) of the total OGs belonged to Class2 (shared) and only a minor fraction of OGs were Class1 (*R. fus*-specific).

Next, to account for the predacious phenotype of *R. fuscipes*, we will test if there is any enrichment of diet-related genes in *R. fus*-specific genes. Among the 21,170 total OGs, we found 926 (4.37%) as diet-related OGs according to our previously defined gene set (method details). These OGs were mainly enriched in pathways relevant to digestion, chemosensory, and detoxification (Figure 2B; Table S7), determining multiple aspects of host’s diet spectrum. We found that Class1 *R. fus*-specific OGs had significantly higher fractions of diet-related functions (13.3% for “*R. fus*-unique”, 14.8% for “*R. fus*-more”, and 14.0% for pooled Class1) compared to Class2 shared OGs (2.0% for the few gradient OGs, 4.2% for other OGs, and 4.2% for pooled Class2) (Figure 2C). For Class1 (*R. fus*-specific), there were totally 47 diet-related OGs (47/335 = 14.0%), significantly higher than the genome-wide baseline (4.37%). This indicates that from the aspect of gene numbers, *R. fuscipes* is genetically different from the two non-predaceous true bugs *A. lucorum* and *R. pedestris* which potentially explains their phenotypic divergence. To provide more information about the difference between the predaceous and non-predaceous species, we performed GO and KEGG analysis of the 23 (13.3% of 173) diet OGs belonging to *R. fus*-unique, 24 (14.8% of 162) diet OGs belonging to *R. fus*-more, and 47 (14.0% of 335) diet OGs as a whole belong to *R. fus*-specific. The results show that these genes were indeed enriched in detoxification, digestion, and perception (Figure S3).

### Diet-related genes in *R. fuscipes* generally had higher expression and diversity

Apart from the genetic divergence among different species, we wonder whether the biological traits could be explained by molecular mechanisms at other layers. We calculated the gene expression profiles from the transcriptome data of three true bug species. For the genes in Class1 *R. fus*-specific OGs, the diet-related genes had significantly higher expression levels than non-diet genes (Figure 2D). This trend further supports the notion that the *R. fus*-specific genes, especially those diet-related ones, are playing an essential role in governing the phenotypic uniqueness of *R. fuscipes* compared to the two non-predaceous bugs. Then, for Class2 genes which are shared by three species, the diet-related genes showed significantly higher expression level in *R. fuscipes* than other two true bugs (Figure 2D). This further suggests that even for the shared genes among three species, *R. fuscipes* still showed strong quantitative difference with non-predaceous bugs on the diet genes. This pattern again bridges the gap between genotype and phenotype. Then, although the non-diet genes of Class2 showed the highest expression in *R. fuscipes*, the differences across three species were obviously weaker than what we observed for diet-related genes (Figure 2D). These observations indicated that the diet-related genes are more likely to explain the phenotypic divergence between predaceous and non-predaceous bugs and that the non-diet genes only play minor roles in the inter-species divergence. Notably, theory of genome evolution dictates that the species-specific genes usually have lower conservation level and expression level than the shared genes across multiple species. This phenomenon is clearly reflected in our data by comparing the gene expression profiles between Class1 and Class2 genes (Figure 2D). Class2 genes were more conserved and therefore had higher expression levels than Class1 genes.

Another salient pattern is, among the Class1 (*R. fus*-specific) genes, those diet-related genes possessed significantly more exons than the non-diet genes (Figure 2E). Accordingly, diet-related genes were longer (Figure S4). It is conceivable that more exons of a gene usually result in more combinations of the spliced transcript and thus more protein isoforms (as revealed by genome annotation and Iso-Seq results, see Figure S5). This fact that diet-related genes in Class1 had more exons than non-diet genes indicated that those diet genes in *R. fuscipes* were more likely to be diversified by alternative splicing. This assumption is further supported by our comparison among Class2 genes, where we found that the diet-related genes in Class2 had more exons in *R. fuscipes* compared to other two non-predaceous true bugs (Figure 2E). In contrast, the non-diet genes in Class2 did not show such strong difference across three species (Figure 2E). These observations raise a possibility that the diet genes in *R. fuscipes* were specifically diversified by alternative splicing, facilitating the formation and flexible regulation of the predaceous-related phenotype.

### Abundant A-to-I RNA editing sites were detected in three true bugs

**Figure 3:**
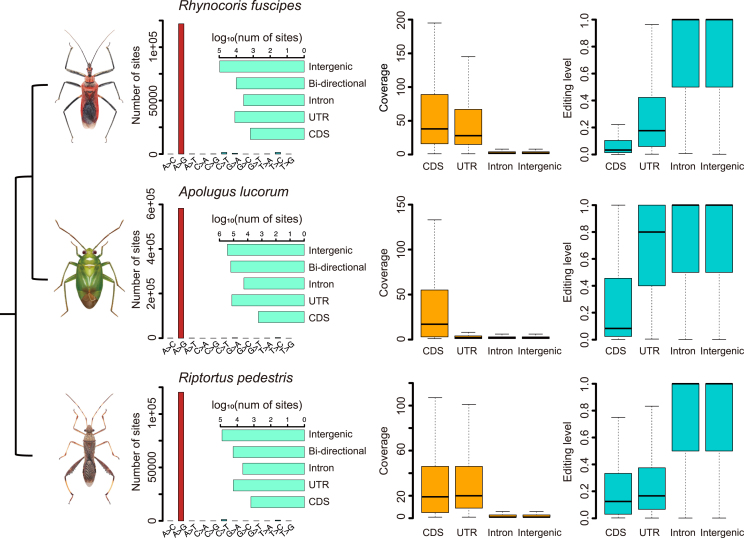
A-to-I RNA editomes of three true bugs Left panel: fractions of variations identified in the transcriptomes. A-to-G variations were dominant and were regarded as A-to-I RNA editing sites. Annotation of A-to-I editing sites was shown as barplots. Middle panel: sequencing coverage on different categories of RNA editing sites. Right panel: editing levels of different categories of RNA editing sites.

To understand to what extent the diet-related genes in *R. fuscipes* are expanded and diversified beyond the genome, we continue to seek for other *cis*-regulatory mechanisms that could diversify the transcriptome or proteome. A-to-I RNA editing is a typical example of post-transcriptional modification that diversifies the transcriptome in a flexible manner.^36^^,^^37^^,^^38^ We sequenced the transcriptome of whole body of *R. fuscipes* and downloaded the comparable transcriptomes of *A. lucorum* and *R. pedestris* from NCBI. We systematically identified A-to-I RNA editing events in three species (method details). By mapping the transcriptomic reads to the reference genomes, we identified as many as 10^5^∼10^6^ RNA-DNA differences (RDD) in three species. Strikingly, among these RDD variations, >96% of them were A-to-G, representing super-strong signals of A-to-I RNA editing (Figure 3). Totally, 121,621 A-to-I RNA editing sites were obtained in *R. fuscipes* after excluding the single nucleotide polymorphisms (SNPs) from the genome resequencing data (and the number was 121,888 before see STAR methods for detail), 582,961 editing sites were obtained in *A. lucorum*, and 120,294 editing sites were obtained in *R. pedestris* (Figure 3). Not surprisingly, the majority of the A-to-I editing sites (>75%) were located in intergenic or intronic regions and only ∼10^3^ sites (<2%) were located in CDS region (Figure 3). This distribution echoes the pattern observed in other insects like bumblebee where hyper-editing sites were highly abundant and were enriched in genomic repeats.^39^ Accordingly, we found that intronic or intergenic editing sites were significantly enriched in repeats while CDS editing sites were depleted in repetitive elements (Figure S6). Notably, we also annotated a considerable fraction of editing sites located in bi-directional transcription regions (where both strands had transcripts). However, in bi-directional regions, A-to-I editing on one strand might come from the T-to-C mutation in the opposite strand, making it technically unreliable (see STAR methods for details). Therefore, this type of editing sites was not included in the following analyses.

To get a clear picture on the functional and evolutionary features of RNA editing sites, we investigated the editing levels of each functional category. CDS editing sites had the highest sequencing coverage and the lowest editing level compared to non-coding editing sites (Figure 3). This is expected because most hyper-editing sites in introns or intergenic regions were covered by a few reads with multiple editing sites on each,^40^ resulting in a low coverage and high editing level on these non-coding sites. For CDS editing sites, nonsynonymous sites did not show higher editing levels than synonymous sites (Figure S6), suggesting that the overall nonsynonymous editing was not favored by natural selection. However, apart from nonsynonymous editing, the transcriptome could also be diversified by intronic editing that affects alternative splicing,^41^^,^^42^^,^^43^ not necessarily edited in coding regions.^26^ For instance, the abundant non-coding editing sites in repetitive elements (like human *Alu*) would create numerous combinations of dsRNA sequences. These edited dsRNAs prevent MDA5 from activating unnecessary immune response.^44^ Therefore, in our data, the prevalent RNA editing in intergenic or intronic regions might also exert a transcriptomic diversification role.

We have found that the *R. fus*-specific diet genes had multiple “advantageous” *cis* features like high expression and more exons that might be related to its phenotypic divergence from the non-predaceous bugs (Figure 2). Here, since the abundant A-to-I RNA editing events would largely diversify the transcriptome, we then aimed to look for the interplay between RNA editing and diet-related genes. First, we looked at the editing sites in genic regions. If RNA editing is enriched in diet genes in *R. fuscipes*, then it will add another layer to diversify those genes and explain the predaceous behavior of *R. fuscipes*.

**Figure 4:**
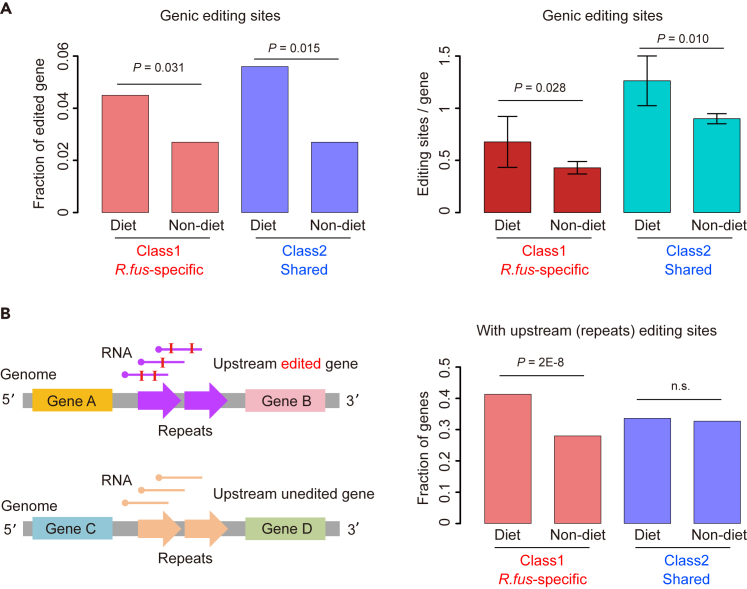
The interplay between A-to-I RNA editing sites and diet-related genes in *R fuscipes* (A) Left panel: fraction of edited genes in each category. *p* values were calculated by Fisher’s exact test. Right panel: number of editing sites per gene. *p* values were calculated by Wilcoxon rank sum tests. (B) Fraction of genes with upstream intergenic region edited. *p* values were calculated by Fisher’s exact test. n.s., not significant.

For the Class1 (*R. fus*-specific) and Class2 (shared) genes, we divided them as diet and non-diet genes as we have done in Figure 2. Then, we calculated the fraction of edited genes in each category. We found that in both classes, diet genes had significantly higher tendency to be edited than non-diet genes in *R. fuscipes* (Figure 4A). Moreover, the average number of editing sites per gene was also significantly higher in diet genes than non-diet genes (Figure 4A). These results suggest that there is an enrichment between A-to-I RNA editing and diet genes in *R. fuscipes*, serving as a mechanism to diversify the transcriptome of diet genes. Together with the other *cis* features like high expression and more exons, these *R. fus*-specific diet genes might facilitate *R. fuscipes* to adapt to its predaceous behavior and related diets.

Next, we switched our attention to the large amount of intergenic editing sites. Interestingly, a recent paper has reported that the human *Alu* elements transcribed from the promoter and enhancer regions would form RNA duplexes and strongly upregulate the expression of downstream genes.^45^ It is known that the human *Alu* RNAs are heavily edited under normal conditions.^46^ Similarly, we intuitively came up with the assumption that the non-coding intergenic repeats in *R. fuscipes*, which were also extensively edited (Figure 3; Figure S6), might function as the regulator of the expression of neighboring (downstream) genes. We divided all genes into two groups according to whether their upstream repeat region had editing sites detected (Figure 4B). We found that the upstream-edited genes were significantly enriched in diet genes for Class I (*R. fus*-specific genes), but not for Class II (Figure 4B). If the upstream editing sites promote gene expression, then this result perfectly echoes our observation that among Class I genes, diet genes had higher expression than non-diet genes, but among Class II genes, diet and non-diet genes showed similar expression in *R. fuscipes* (Figure 2D).

In this part, we found that both genic and intergenic editing sites were enriched for diet genes among the Class I (*R. fus*-specific) genes. While genic editing sites might diversify the host genes, the intergenic editing sites might play a promoting role of downstream gene expression. These *R. fus*-specific diet genes are likely to contribute to the predaceous phenotype of *R. fuscipes*.

### Collaboration of multiple *cis*-regulatory mechanisms that diversifies the diet genes in *R. fuscipes*

**Figure 5:**
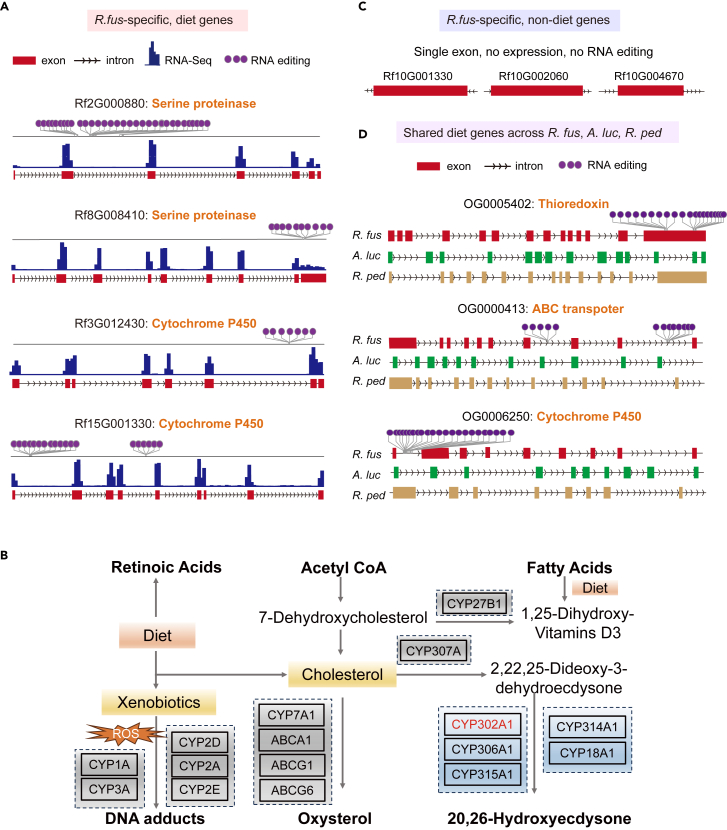
Illustration of diet genes (*R. fus*-specific or shared) with high expression, many exons, and abundant A-to-I RNA editing sites (A) Gene models of *R. fus*-species diet genes. The expression levels were represented by sequencing coverage plot. (B) An example of a pathway containing an *R. fus*-specific diet gene belonging to the P450 family. This pathway functions in lipid metabolism and toxicity. (C) *R. fus*-species non-diet genes. The displayed examples have single exons, no expression, and no RNA editing sites. (D) Shared diet genes across *R. fuscipes*, *A. lucorum*, and *R. pedestris*. The exon/intron lengths were not exactly proportional to the real lengths. However, longer exons/introns were still longer in the diagram.

To fully illustrate the enrichment of multiple *cis*-regulatory mechanisms in diet genes in *R. fuscipes* we selected several Class1 (*R. fus*-specific) and Class2 (shared) diet genes that had high expression, a plenty of exons, and abundant RNA editing sites in *R. fuscipes*. From the multiple genes meeting these criteria, we selected a few examples of well-acknowledged genes participating in shaping the diet traits of animals. For the Class1 *R. fus*-specific genes, we displayed diet-related genes Rf2G000880 and Rf8G008410 (serine proteinase activity) and Rf3G012430 and Rf15G001330 (cytochrome P450 activity) (Figure 5A). Serine proteinases exert a wide range of molecular and cellular functions such as protein digestion, degradation, and cellular and humoral immunity.^47^^,^^48^^,^^49^ Cytochrome P450 plays a crucial role in the synthesis of essential macromolecules necessary for biological survival, concerned with the oxidative detoxication of phytoalexins, drugs, and other xenobiotics.^50^ For example, gene (CYP302A1) belongs to the P450 family and this gene only exists in *R. fuscipes*. CYP302A1 participates in the metabolic pathways for the nuclear receptors in lipid metabolism and toxicity (Figure 5B) and this pathway might account for the predacious features of *R. fuscipes*. In fact, all genes shown in Figures 5A and 5B are closely related to host’s diet spectrum. Accordingly, these genes also had high expression, extensive RNA editing, and multiple exons in *R. fuscipes* and therefore were very likely to contribute to the predaceous phenotype unique to *R. fuscipes*. In contrast, the *R. fus*-specific non-diet genes were of less exons, lowly expressed, and few RNA editing sites (Figure 5C).

For the Class2 genes (shared genes), we displayed the gene models of diet-related genes (thioredoxin, ABC transporter, and cytochrome P450) in three species (Figure 5D). Thioredoxin serves important functions in cell signaling defense against oxidative damage and stress response,^51^^,^^52^ and ABC transporter is responsible for xenobiotic transport and detoxification.^53^^,^^54^ These chemosensory and detoxification-related genes would also contribute to the diet formation of hosts. Notably, although these diet-related genes existed in all species, some *cis*-regulatory mechanisms like RNA editing only existed in *R. fuscipes* (Figure 5D). Together with the divergence in gene regulation (like the expression and splicing patterns) between *R. fuscipes* and other two species, it is likely that these diet genes might also play a role in shaping the phenotypic divergence between predaceous and non-predaceous bugs.

### *R. fuscipes*-specific diet genes were overrepresented in predaceous true bugs: From a larger evolutionary scale

We previously only selected *A. lucorum* and *R. pedestris* as representative non-predaceous true bugs due to their high-quality genomes and matched transcriptomes. Here, we extended our target species to the whole Hemiptera clade plus an outgroup in Thysanoptera. Our goal is to re-confirm the patterns found in *R. fuscipes*: to verify whether the *R. fus*-specific diet genes truly contribute to the diet shift during the evolution of true bugs or other closely related clades. By requiring the availability of genome sequence coupled with annotation file, we totally selected 33 species of Hemiptera and an outgroup *Thrips palmi* (Table S8: *R. fuscipes* and additional 32 species), including 5 predaceous bugs and 28 phytophagous bugs. For the 47 diet-related OGs in Class1 (*R. fus*-specific OGs), we scanned the protein sequence against the 33 reference genomes to obtain the number of genes in each OG of each species.

**Figure 6:**
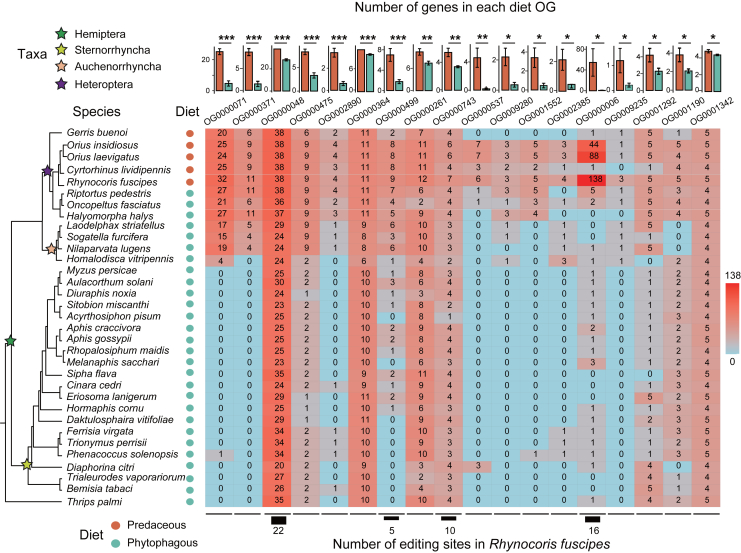
Comparison of diet-related gene numbers in 32 hemipteran species and an outgroup Among the *R. fus*-specific (Class1) diet OGs, 18 of them showed significant difference in gene numbers between predaceous and non-predaceous species. The phylogenetic tree of the 33 species was shown on the left. The detailed taxonomy of all species was provided in Table S8. The gene number of each OG was labeled for each species in the heatmap. Barplots on the top showed the mean (±S.E.M.) of gene numbers in predaceous versus phytophagous species for each OG. *p* values were calculated by T test on the gene numbers with log_2_(n+1) transformation. ∗, *p* < 0.05; ∗∗, *p* < 0.01; ∗∗∗, *p* < 0.001.

We first found that the 5 predaceous species did not form a monophylic group but were located at relatively recent branches (Figure 6). Together with the fact that in some other Heteroptera species, predaceous groups might have phytophagous sister groups,^10^ it could be inferred that the predaceous phenotype has independently emerged during Heteroptera evolution. We then compared the number of genes in each group between predaceous and phytophagous species. Strikingly, we found that 18 (38.3%) out of 47 diet OGs showed significantly higher numbers of genes in predaceous species than in phytophagous species (Figure 6). In contrast, no OGs had more genes in phytophagous species than in predaceous OGs. This difference (18/47 versus 0/47) was significant under Fisher’s exact test (*p* = 5.1E-7). The results suggest that the *R. fus*-specific diet genes identified by us might play a role in the phenotypic divergence of predaceous bugs. Notably, although these 18 OGs showed significant difference in gene numbers between 5 predaceous versus 28 phytophagous species, they were not necessarily absent in all phytophagous species (Figure 6). This suggests that (1) phenotype is not only decided by the presence/absence of particular genes, it might also be quantitatively determined by gene numbers; (2) moreover, it is possible that in predaceous species like *R. fuscipes*, these 18 diet OGs possess other transcriptional regulatory mechanisms (like gene expression, splicing, and RNA editing) to further diversify the gene functions and lead to the predatory phenotype.

**Figure 7:**
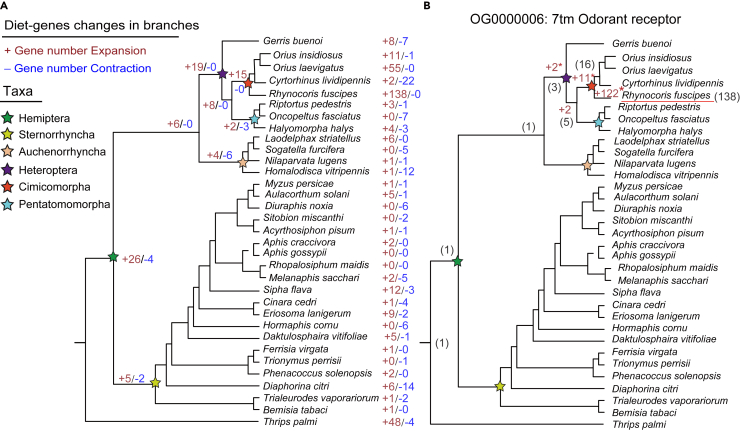
The expansion and contraction of diet-related Class1 OGs in 32 hemipteran species and an outgroup (A) Evolutionary summary of all diet genes shown in Figure 6. The numbers of expanded genes were in red. The numbers of contracted genes were in blue. (B) Evolutionary history of genes in OG0000006 (7tm Odorant receptor). ∗ represents significant expansion.

Next, we refined this analysis in a phylogenetic context (Figure 7A). For the gene numbers of diet-related OGs we obtained for each species (Figure 6), we deduced the expansion and contraction of each OG at each node in the tree (see STAR methods for detail). Not surprisingly, in the clades containing predacious bugs (like Heteroptera and Cimicomorpha), diet-related genes were expanded at the corresponding node (Figure 7A). At the end of each branch, apart from *R. fuscipes* itself, diet genes were also dramatically expanded in species like *Orius insidiosus* and *Orius laevigatus*. Specifically, we illustrate the evolutionary gains and losses of genes in OG0000006 (7tm Odorant receptor) as an example (Figure 7B). This gene family senses and responds to many odors and should be closely connected to the predacious behavior. The evolutionary trajectory shows that this gene family was continuously expanding from the ancestral node of predacious bugs to the branch leading to *R. fuscipes*, but its copy number remained un-elevated before the emergence of predacious bugs (Figure 7B). This is the best evidence to prove the connection between the diet-related genes and the predacious phenotype.

## Discussion

We have sequenced and assembled the first chromosome-level genome of predatory true bug *Rhynocoris fuscipes* and made plausible explanations on its predatory nature in the light of genome evolution. From multiple layers of genomic and transcriptomic regulation, we found solid observations that diet-related genes are overrepresented in predacious species compared to non-predacious species. Our work is of great significance to various fields. On one hand, since *R. fuscipes* has a broad spectrum of preys, it is a promising biocontrol agent to restrict multiple pests in the fields. Understanding its genomic features might benefit the mass-rearing of *R. fuscipes*. From a broader sense of fundamental biological principles, our findings would advance our understanding on the relationship between genotype and phenotype, that is, how central dogma determines organisms’ phenotype.

DNA variations are the major source of phenotypic innovation and adaptation. However, diversity and adaptation could also be achieved at post-transcriptional or epigenetic levels such as alternative splicing, RNA editing, and expressional regulation. In theory, the observed phenotypic innovations (such as predation in true bugs) might come from either the DNA level or RNA level or both. However, in some cases, genome evolution and transcriptome diversification are mutually exclusive^55^ due to the fact that many types of transcriptional regulation/modifications require particular sequence context such as the HAG motif for A-to-I RNA editing (H = non-G), the RRAC motif for m^6^A methylation (R = A or G),^56^ and the GU-AG rule for alternative splicing.^33^ The strict sequence context is the prerequisite for the occurrence of these *cis*-regulatory mechanisms. Therefore, in order to maintain the flexible regulation at RNA level, the DNA sequence context must be highly conserved. This largely restricts genome evolution. The trade-off between transcriptome diversity and genome evolution in cephalopods is a typical example.^36^^,^^55^

Interestingly, in *R. fuscipes*, we found a general trend that both genomic features and transcriptomic regulatory mechanisms might collaboratively contribute to the predatory phenotype: (1) the *R. fus*-specific genes were enriched in diet genes, suggesting that the presence/absence of particular genes might shape the phenotypic divergence between different species; (2) the transcriptomic regulations like gene expression, alternative splicing, and RNA editing also showed enrichment in diet genes in *R. fuscipes*, adding another layer to transcriptome complexity and might account for the relevant phenotype as well. Moreover, the primary conclusion based on three species that “diet genes were overrepresented in predaceous bug *R. fuscipes*” was further verified in a larger evolutionary scale involving 32 hemipteran species and an outgroup. Many of the diet-related OGs showed significantly higher gene numbers in predaceous species compared to phytophagous species.

Diet-shift frequently occurred during insect evolution. Understanding the genetic basis behind this transition would help us better govern these insect resources to benefit ourselves, such as the rearing and releasing of natural enemy species. Our current work has first utilized a small size of high-quality genomes to accurately identify the *bona fide* differential genes between predaceous *R. fuscipes* and other two non-predaceous species, and then generalized our findings to a much broader range of species (with lower-quality genomes). Thus, a limitation here is the lack of high-quality genomes for the ∼30 public hemipteran species. There is no sister species of predacious and phytophagous reduviids, which should be useful for studying genomic evolution associated with dietary change. Nevertheless, we still obtained expected results that the *R. fus*-specific diet genes were overrepresented in predaceous species. In the future, when more high-quality genomes and transcriptomes of hemipteran species are available, we may accurately discover the gene families specific to predaceous species and then perform deeper analysis and verification on the genetic determinant of diet-shift in insects.

### Limitation of the study

We only used two additional Hemiptera species for the main comparative genomic analysis due to the limited availability of high-quality genome assembly in the public database. This might miss some true positive target genes responsible for diet-shift. Nevertheless, we still found an enrichment of diet-related genes in *R. fuscipes*, suggesting the overall reliability of our findings. For the transcriptome analysis, the RNA-seq from whole bug was used. Ideally, the transcriptomes from salivary glands are favorable for analyzing the diet-related genes, and the transcriptomes from heads or nervous systems are favorable for analyzing A-to-I RNA editing. But we indeed found several interesting patterns albeit the signals might have been diluted. These details could be improved in future works.

## STAR★Methods

### Key resources table

**Table:** 

REAGENT or RESOURCE	SOURCE	IDENTIFIER
**Biological samples**		

adults of *Rhynocoris fuscipes*	Kunming, Yunnan Province, China	N/A

**Critical commercial assays**		

Blood and Cell Culture DNA Midi Kit	QIAGEN	Cat# 19060

**Deposited data**		

Genome sequencing and assembly data	GenBank	JAPXFL000000000.1
PacBio long-read sequencing data	SRA database	SRR22799260
Hi-C data	SRA database	SRR22799256
Illumina short-read sequencing data	SRA database	SRR22799257
transcriptome data	SRA database	SRR22799258; SRR22799259

**Experimental models: Organisms/strains**		

*Rhynocoris fuscipes*	Kunming, Yunnan Province, China	N/A

**Software and algorithms**		

Fastp v0.21.0	Chen et al.^57^	N/A
JELLYFISH v2.1.3	Marcais & Kingsford^58^	N/A
GenomeScope v2.0	Vurture et al.^59^	N/A
HiCanu v2.0	Nurk et al.^60^	N/A
Purge_dups v1.2.3	Guan et al.^61^	N/A
BWA-MEM v0.7.17	Li and Durbin^62^	N/A
Juicer v1.5	Durand et al.^63^	N/A
3D-DNA pipeline	Dudchenko et al.^64^	N/A
BUSCO v5.2.2	Simao et al.^65^	N/A
Merqury v1.1	Rhie et al.^66^	N/A
EDTA v1.9.4	Ou et al.^67^	N/A
RepeatMasker v4.0.7	Chen et al.^68^	N/A
RepeatModeler v2.0.1	Jurka et al.^69^	N/A
TANDEM REPEATS FINDER v4.07b	Benso et al.^70^	N/A
IsoSeq v 3.4.0	N/A	https://github.com/PacificBiosciences/IsoSeq
HISAT2 v2.2.1	Kim et al.^71^	N/A
StringTie v2.4.0	Kovaka et al.^72^	N/A
Exonerate v2.4.0	Slater & Birney^73^	N/A
AUGUSTUS v3.2.3	Stanke et al.^74^	N/A
MAKER v2.31.10	Cantarel et al.^75^	N/A
eggnog-mapper v2.0.1	Cantalapiedra et al.^76^	N/A
InterProscan v5.0	Jones et al.^77^	N/A
BLAST v2.2.28	Camacho et al.^78^	N/A
HMMER v3.3.2	Finn et al.^79^	N/A
JCVI v1.1.17	Tang et al.^80^	N/A
OrthoFinder v2.5.4	Emms & Kelly^81^	N/A
MAFFT v7.487	Katoh & Standley^82^	N/A
PHYKIT v1.11.7	Steenwyk et al.^83^	N/A
STAR v2.7.6a	Dobin et al.^84^	N/A
FeatureCounts v.0.3	Liao et al.^85^	N/A
TRIMAL v1.4	Capella-Gutiérrez et al.^86^	N/A
FASCONCAT-G v1.0.4	Kück & Longo^87^	N/A
BACOCA v1.1	Kück & Struck^88^	N/A
IQTREE v2.2.0	Nguyen et al.^89^	N/A
CAFÉ v5	Bie et al.^90^	N/A
Minimap2	Li et al.^91^	N/A
SPRINT v0.1.8	Zhang et al.^92^	N/A
SnpEff v4.5	Cingolani et al.^93^	N/A

**Other**		

bash code and R script	N/A	https://doi.org/10.6084/m9.figshare.25917973.v1

### Resource availability

#### Lead contact

Further information and requests can be directed to Prof. Hu Li (tigerleecau@hotmail.com).

#### Materials availability statement

This study did not generate new unique reagents.

#### Data and code availability

• The chromosome-level genome assembly sequence is available at NCBI (https://www.ncbi.nlm.nih.gov/) GenBank through accession number JAPXFL000000000.1. The PacBio long-read sequencing data have been deposited in the NCBI SRA database under accession number SRR22799260. The high-quality Hi-C data are available through the NCBI SRA database under accession number SRR22799256. The high-quality Illumina short-read sequencing data are available in NCBI SRA under accession number SRR22799257. The transcriptome data are available in NCBI SRA under accession numbers SRR22799258 and SRR22799259. The genomes of additional 32 species of Hemiptera and an outgroup were downloaded from NCBI (https://www.ncbi.nlm.nih.gov/, also see Table S8 for the links).
• Code availability. Most of the analyses were done by well-acknowledged software and the various software have been properly cited in the manuscript. We have submitted the bash code and R script to FigShare with accession link https://doi.org/10.6084/m9.figshare.25917973.v1.
• Any additional information required to reanalyze the data reported in this paper is available from the lead contact upon request.

### Experimental model and study participant details

*Rhynocoris fuscipes* adults were collected in Kunming, Yunnan Province, China (102.80°E, 24.96°N), and reared in the lab with 4-5th instar larva of *Spodoptera litura* and kept at 25 ± 1°C, 70 ± 5% relative humidity, and 14 h light: 10 h dark. The population was maintained in lab for six months (five generations) before the specimens were collected for sequencing. Random mating within a population is not feasible for *R. fuscipes* due to their nature of “hunting/eating each other”. Therefore, in each generation, the insects were subjected to one-to-one mating artificially. However, we did not construct an iso-female or inbreeding line to pursue a clean genetic background due to inbreeding depression. Instead, the offspring from a one-to-one mating pair was then hybridized with the offspring from another one-to-one mating pair. Since the original population is not huge and the individuals used for sequencing came from the same generation, they had a relatively close genetic background. This will reduce the risk of false-positive RNA editing to some extent (see the following sections). The samples used in this study were adults of *R. fuscipes*, and their sex was determined prior to subsequent experiments. For detailed information on the sex of the samples for each experiment, see the method details. Then, samples were quickly placed into collection tubes, flash-frozen in liquid nitrogen, and stored at −80°C until usage.

### Method details

#### Genome and transcriptome sequencing of *R. fuscipes*

Up to four female adults were enough for PacBio sequencing. Genomic DNA of four individuals was extracted using the CTAB method, followed by purification using a Blood and Cell Culture DNA Midi Kit (QIAGEN, Germany). Extracted DNA purity and concentration were determined with 0.75% agarose gel electrophoresis and a Qubit 2.0 Fluorometer (Thermo Fisher Scientific, USA), respectively. A long-fragment library with an insertion size of ∼15 kb was performed and generated SMRT PacBio High-Fidelity (HiFi) reads using the Pacific Bioscience sequel II system (Pacific Biosciences). The same set of DNA was subjected to library construction with 150 bp paired-end short reads and sequenced on Illumina Novoseq 6000 platform. This next generation sequencing of the DNA was referred to whole genome sequencing (WGS) for simplicity.

A Hi-C library was constructed using one *R. fuscipes* female adult. The sample was cross-linked with a 2% formaldehyde isolation buffer and then treated with DpnII (NEB) to digest nuclei. Biotinylated nucleotides were used to repair the tails, and the ligated DNA was split into fragments of 350 bp in length. Hi-C library was sequenced on an Illumina Novoseq 6000 platform.

A pooled *R. fuscipes* sample was prepared including one male and one female. Total RNA was extracted with TRIzol reagent (Thermo Fisher Scientific, USA). A paired-end library was constructed using the TruSeq RNA Library Preparation Kit (Illumina, USA) and sequenced on an Illumina Novoseq 6000 platform. Total RNA (1 μg) was used to construct a full-length transcript isomer library using the SMRT bell Express Template Prep Kit 2.0 (Pacific Biosciences, USA). Target-size sequences were generated using the PacBio sequel II platform.

#### Genome assembly and annotation of *R. fuscipes*

Illumina short reads from *R. fuscipes* were filtered by Fastp v0.21.0 software with default parameters.^57^ Genome heterozygosity, repeat content, and genome size were determined with a Kmer-based statistical analysis using JELLYFISH v2.1.3^58^ and GenomeScope v2.0.^59^

A draft genome was assembled by HiCanu v2.0 with default parameters.^60^ The Purge_dups v1.2.3^61^ software was then applied to remove heterozygous duplication and improve continuity. A scaffolding pipeline based on^63^ was used to generate a high-quality chromosome-scale genome. After filtering adapter and low-quality sequences by Fastp v0.21.0,^57^ Hi-C data were mapped to the contig genome by BWA-MEM v0.7.17^62^ with the following parameters (mem -SP5M). Next, the DpnII site was generated using the script ‘generate_site_position’ in Juicer v1.5.^63^ After scaffolding, ordering, and clustering the peregrine contigs via 3D-DNA pipeline (-r 2),^64^ the chromosome-scale genome was performed.

We evaluated the completeness of the chromosomal-level genome using Benchmarking Universal Single-Copy Orthologs (BUSCO v5.2.2) under the insecta_odb10.^65^ Furthermore, BWA-MEM v0.7.17 and Merqury v1.1^66^ were used to assess the accuracy and base error of *R. fuscipes* genome.^62^

Transposable elements (TEs) were annotated in Extensive *de novo* TE Annotator (EDTA) v1.9.4.^67^ And RepeatMasker v4.0.7 and RepeatProteinMasker v4.0.7 (engine WUBLAST)^68^ were applied for identifying repeat sequences based on RepBase edition 20170127.^69^ Besides, repeats were masked with *de novo* predictions using RepeatModeler v2.0.1. Tandem repeats were annotated using TANDEM REPEATS FINDER v4.07b.^70^

Genes in the assembled genome were predicted with a combination of homology-based, transcriptome-based, and *de novo* methods. (1) Homology-based predictions involved downloaded homologous proteins and transcripts from *Apolygus lucorum*, *Cimex lectularius*, *Orius laevigatus*, *Rhodnius prolixus*, *Triatoma rubrofasciata* (covering all available species in Cimicomorpha), and model insect *Drosophila melanogaster* (NCBI, https://www.ncbi.nlm.nih.gov/; InsectBase v 2.0). The IsoSeq v 3.4.0 workflow was used to generate high-quality full-length transcripts with quality parameters of 0.99 (https://github.com/PacificBiosciences/IsoSeq). (2) For transcriptome-based methodology, RNA-seq data were mapped to the reference genome using HISAT2 v2.2.1^71^ and assembled into transcripts using StringTie v2.4.0.^72^ Homologous proteins and transcripts were then aligned using Exonerate v2.4.0 for training gene sets.^73^ Meanwhile, a sorted and mapped bam file of RNA-seq data were transferred to a hint file using the bam2hints program in AUGUSTUS v3.2.3.^74^ (3) Self-trained sets were combined with hint files as inputs for AUGUSTUS to predict *de novo* coding genes from the assembled genome. Finally, the homology-based, transcriptome-based, and *de novo* results were merged in MAKER v2.31.10 to generate a high-confidence gene set.^75^

Gene structure and annotations were determined using eggnog-mapper v2.0.1,^76^ InterProscan v5.0,^77^ BLAST v2.2.28,^78^ and HMMER v3.3.2^79^ to search against NCBI non-redundant protein (Nr), Gene Ontology (GO), Clusters of Orthologous Groups of Proteins (COG), Kyoto Encyclopedia of Genes and Genomes (KEGG), Swiss-Prot, and Pfam.

#### Synteny analysis and the determination of sex chromosome in *R. fuscipes*

We analyzed the synteny between *Rhynocoris fusipes* and other hemipteran species using JCVI v1.1.17 with default parameters^80^ (Figure S7). Since we did not separately sequence the female and male genomes, we can only manage to determine the sex chromosome by comparing the synteny between *Rhynocoris fusipes* and other hemipteran species (Figure S7).

First, we confirmed that the *R. fusipes* genome has sex chromosomes, with a karyotype of *N* = 12A + X1X2X3.^94^ To determine which chromosomes in our genome assembly are the sex chromosomes, we collected the chromosome-level genomes of additional three hemipteran species which are known to possess sex chromosomes. Apart from *A. lucorum* and *R. pedestris*, we chose a closely related hematophagous hemipteran *Triatoma rubrofasciata*. Particularly, the single X chromosome in the *R. pedestris* genome has already been determined,^35^ The karyotypes of *A. lucorum*, *R. pedestris* and *T. rubrofasciata* are *N* = 16A + X,^34^ N = 6A + X,^35^ and *N* = 11A + X1X2,^95^ respectively. Synteny analysis showed that the Chr13, 14 and 15 of *R. fusipes* exhibited high homology with Chr12 and 13 of *T. rubrofasciata*, Chr1 of *A. lucorum*, and the ChrX of *R. pedestris* (Figure S7). The numbers of sex chromosomes in each species are consistent with the reported karyotypes. Thus, we conclude that the Chr13, 14, 15 in *R. fusipes* correspond to the X1, X2 and X3 chromosomes.

#### The selection of high-quality genomes of non-predaceous true bugs

To select the high-quality genomes of non-predaceous true bugs as a control, we required multiple criteria involving: (1) a chromosome level genome with scaffold N50 > 40 Mb; (2) with gene annotation file; (3) with a matched transcriptome of whole bug. We finally focused on two non-predaceous true bugs *Apolygus lucorum* (*A. lucorum*) and *Riptortus pedestris* (*R. pedestris*). *A. lucorum* is mainly phytophagous (sometimes omnivorous) while *R. pedestris* is strictly phytophagous. The genome sequences of *A. lucorum*^34^ and *R. pedestris*^35^ were obtained from InsectBase 2.0 database (http://v2.insect-genome.com/Organism/85; http://v2.insect-genome.com/Organism/689), and the RNA-seq data were collected from NCBI SRA under accession numbers SRR10411315 and SRR21672250. Both datasets were sequenced on the illumina platform.

#### Ortho-group (OG) identification in *R. fuscipes*, *A. lucorum*, and *R. pedestris*

We created ortholog groups from the genomes of *R. fuscipes*, *A. lucorum* and *R. pedestris*. The genome sequences were downloaded from InsectBase 2.0 database (http://v2.insect-genome.com/Organism/85; http://v2.insect-genome.com/Organism/689), and the longest protein and transcript sequences of each gene were kept. OrthoFinder v2.5.4 was used to identify orthologous and paralogous genes across these species, utilizing BLAST all-vs-all searches and clustering genes based on their similarity. The parameters ‘-S blast -M msa -T fasttree’ were applied to ensure accurate grouping.^81^ Functional annotation of proteins was carried out via InterProScan v5.0^77^ against GO, InterPro, and PFAM databases. Ortho-groups (OGs) were analyzed using KinFin v1.0 by providing function annotation. In the next step, these OGs were further used for defining the OGs involved in feeding habits (the diet-related genes).

Notably, here we would like to distinguish two terms “genes” and “ortho-groups”. For example, 17,486 genes were annotated in the *R. fuscipes* genome, together with the genes annotated in the *A. lucorum* and *R. pedestris* genome, all these gene were collected and input to OrthoFinder. The software will group these genes according to their similarity. Totally, 21,170 ortho-groups (OGs) were found among three species: (1) One OG must contain at least one gene from any species; (2) One gene can only belong to one OG; (3) One OG can contain more than one gene, and these multiple genes can come from different species (orthologs) or come from the same species (paralogs). Both orthologs and paralogs are included in OGs.

According to these rules, the number of OGs must be theoretically smaller than or equal to the sum of gene numbers in all species. However, in reality, since multiple conserved genes are usually grouped into one OG, the actual number of OGs is much smaller than the sum of gene numbers in all species. Given that *R. fuscipes*, *A. lucorum*, and *R. pedestris* all belong to Hemiptera, it is natural to obtain that each species has 15,000–20,000 genes and the final number of OG is 21,170.

To prove that the classification is convincing, we compared the pairwise identity within each OG and between genes of different OGs. Among all the OGs identified, 9,405 OGs contained more than one gene. First, we calculated the intra-OG pairwise identities of those genes. Then, for the inter-OG identity, we randomly divided the 9,405 OGs into 100 equal bins (with each bin having ∼94 OGs), and randomly selected one gene from each of the 94 OGs. The mean pairwise identity of the 94 genes within each bin was regarded as the inter-OG identity. To calculate the identity between two sequences, MAFFT v7.487^82^ and PHYKIT v1.11.7^83^ were used. The results showed that the intra-OG similarity was significantly higher than the inter-OG similarity (Figure S8).

#### Definition of diet-related OGs

Candidates of diet-related genes were collected by searching previous literatures with keywords.^34^^,^^96^^,^^97^^,^^98^ The terms were involved in detoxification, chemosensory and digestion adaptation. For these gene families, their domain information was downloaded from the Pfam database, including Cytochrome P450 (P450s, PF00067), Glutathione S-transferase (GSTs, PF00043), Carboxylesterases (CCEs, PF00135) and ATP-binding cassette transporters (ABCs, PF00005); Ionotropic receptors (IR, PF00060), Gustatory receptors (GR, PF06151/PF08395), Odorant receptors (OR, PF13853/PF02949), Odorant-binding proteins (OBP, PF01395), Chemosensory proteins (CSP, PF03392) and Sensory neuron membrane proteins (SNMPs, PF01130); as well as Serine protease (PF00089), Serpin (PF00079), Carboxypeptidase (PF00246), Aspartic protease (PF00026), Lipase (PF00151/PF01764/PF06350/PF04083/PF01734/PF00657/PF13472), Alpha amylase (PF00128), Thioredoxin (PF00085), CUB (PF00431) and Ptu family (PF08117).

#### Public transcriptomes of *A. lucorum* and *R. pedestris*

The downloaded transcriptome data used in this study are available in NCBI SRA under accession numbers SRR10411315 (*A. lucorum*) and SRR21672250 (*R. pedestris*). These transcriptomes were produced for whole body, the same design as our *R. fuscipes* transcriptome data, enabling the comparison of gene expression between species. While each SRR ID represents one biosample (library/run), we noticed that there are several parallel replicates of the those biosample SRR10411315 or SRR21672250. In theory we should analyze all other libraries. However, since our *R. fuscipes* transcriptome only contains one library, considering the potential “detection bias” caused by sequencing depth/coverage, in each species we only selected one biosample with the closest data-size to our *R. fuscipes* transcriptome data. Moreover, we did not find the information about the stage or sex of the public samples. For stage, if not mentioned, by default it should be adult. For gender, if not mentioned, by default it should be mixed female and male as we have done with our transcriptome data. But we admit that the precise information is missing and especially the gender is uncertain, which might be a limitation in this study.

Nevertheless, the following two aspects can reduce the bias caused by the uncertainty. (1) Regarding our analysis on A-to-I RNA editing, the fact is that RNA editing is highly robust between two genders. The gender-specific sites, if any, do not affect the overall editing profile of a species. This fact is supported by several RNA editing studies^25^^,^^99^; (2) For the analysis on expression of diet related genes which we focus on, most genes are likely expressed in the common tissues shared by the two genders like the salivary gland, and thus the differentially expressed diet genes between species are unlikely caused by the bias of “uncertain gender”. Moreover, as explained, if not mentioned by the provider, the gender should be mixed female and male as our transcriptome.

#### Calculation of gene expression

RNA-Seq transcriptomes were mapped to reference genome using STAR v2.7.6a with default parameters.^84^ Reads count for each gene was calculated with FeatureCounts v.0.3.^85^ Gene expression was measured by RPKM (reads per kilobase per million mapped base). Only the exonic reads were used to calculate RPKM.

#### Classification of OGs regarding their gene numbers in *R. fuscipes*, *A. lucorum*, and *R. pedestris*

The classification of OGs across *R. fuscipes*, *A. lucorum*, and *R. pedestris* was based on the presence/absence or gene number of each OG in each species.

(1) *R. fus*-unique OGs: *R. fus* > 2 & *A. luc* = 0 & *R. ped* = 0, resulting in 173 OGs.
(2) *R. fus*-more OGs: *R. fus* > 2 × max (*A. luc*, *R. ped*), resulting in 162 OGs.
(3) Gradient OGs: *R. fus* > 1.5×*A. luc* & *A. luc* > 1.5×*R. ped*, resulting in 50 OGs. The reason for defining the “gradient” OGs is that *A. lucorum* is not strictly phytophagous (sometimes omnivorous) so that we reserve the possibility that some genomic features of *A. lucorum* might be the intermediate between *R. fus* and *R. ped.* We did not define gradient OG by “*R. fus* > 2×*A. luc* & *A. luc* > 2×*R. ped*” because this case would be entirely included in the “*R. fus*-specific” category.
(4) The “other” 20,785 OGs.

The *R. fus*-unique and *R. fus*-more groups are collectively defined as “*R. fus*-specific” OGs (Class1, 162 + 173 = 335 OGs). The 50 + 20,785 = 20,835 OGs were classified as Class2, termed “shared OGs”.

Different cutoffs were used to define *R. fus*-unique and *R. fus*-more OGs (Figure S2), and the conclusion remains highly robust. For example, *R. fus*-unique OG was defined as *R. fus* > j & *A. luc* = 0 & *R. ped* = 0, where j = (0, 1, 2, 3, 4, 5, 6) were tried. Obviously, j = 0 and j = 1 were not stringent enough and they resulted in a large number of *R. fus*-unique OGs and a low fraction of diet OGs among them. From j = 2, the fractions of diet OGs became stable (>12%, see Figure S2) and were remarkably higher than the genomic baseline 4.37%. Thus, j = 2 was used in the downstream analysis. Similarly, the most plausible cutoff for *R. fus*-more OGs (k = 2, see Figure S2) was used in the downstream analysis.

#### Identification of diet-related OGs in 32 hemipteran species and an outgroup

To confirm the generality of our findings in a broader range of predaceous and non-predaceous insects, we collected the genomes of additional 32 species of Hemiptera and an outgroup from NCBI (https://www.ncbi.nlm.nih.gov/) (see the corresponding part in the main text for the detailed species). We have already identified 47 *R. fus*-specific diet OGs. We searched the protein sequences of these 47 OGs in the genomes of 33 species using BLAST algorithm tblastn (E-value cut-off of ≤ 1e-5).^78^ Then, each OG had a gene number in each species.

#### Construction of phylogenetic tree

We constructed the phylogenetic tree using orthologous genes with 33 species. In brief, BUSCO sets are defined as collections of near-universal single-copy genes, which are rarely lost or duplicated. We collected 1,367 insect coding genes from database insecta_odb10 in BUSCO v5.2.2.^65^ Orthologous protein sequences of each candidate BUSCO group were aligned using MAFFT v7.487 with auto strategy.^82^ Sequence alignments were trimmed and concatenated by TRIMAL v1.4^86^ and FASCONCAT-G v1.0.4.^87^ To reduce the possible systematic errors in large genomic datasets, we calculated the compositional heterogeneity of loci by BACOCA v1.1.^88^ Next, orthologous groups with single-copy orthologues present in 90% of the species were used for phylogenetic tree using IQTREE v2.2.0 under model identified by ModelFinder (--m MFP).^89^

Diet-related genes expansion and contraction was estimated by CAFÉ v5,^90^ based on maximum likelihood and reduction methods. Topology and branch lengths of phylogenetic tree were considered when inferring the significance of changes in gene number in each branch.

#### Quantification of number of transcripts/isoforms per gene

One gene might have different splicing isoforms coming from the combination of different exons. We used two methodologies to test whether the number of isoforms per gene increases with the number of exons per gene. First, according to the genome annotation file, we directly counted how many different transcript IDs does a gene have and how many exons does this gene have. Then a Spearman correlation was calculated. Second, we used the third-generation transcriptome Iso-Seq data. Using Minimap2 v2.17 with default parameters,^91^ we mapped the Iso-Seq reads to the reference transcript sequences (a file termed “all.mRNA.fasta”). The best mapper of each read was maintained according to the flag score. The reference transcript ID with reads mapped were counted, representing the actually expressed isoforms. Then we calculated how many transcript IDs (with mapped Iso-Seq reads) does a gene have, and correlated this number with the number of exons per gene.

#### Identification and annotation of A-to-I RNA editing sites

For *R. fuscipes*, *A. lucorum*, and *R. pedestris*, A-to-I RNA editing sites were identified using SPRINT v0.1.8.^92^ This software first mapped the RNA-Seq reads to the reference genome, and then called variants in the RNA-Seq. The A>G variation made up of >96% of the total variations, standing for strong evidence for A-to-I RNA editing. Notably, although the software SPRINT claims to be “DNA-free”, which means that it identifies RNA editing sites without the need for DNA-resequencing of the matched sample, there is still the worry that the single nucleotide polymorphisms (SNPs) at DNA level might introduce some false positive variants in the RNA-Seq. Therefore, we used the WGS of our *R. fuscipes* sample to mitigate the effect of SNPs. As we have discussed, although the WGS was not strictly from the same individuals of the RNA-Seq data, the insects were collected from the same generation of a small population reared by one-to-one mating, and therefore the collected specimens had a relatively close genetic background. By mapping the *R. fuscipes* WGS reads to the reference genome with BWA followed by variant calling by GATK, we obtained 31,445,484 SNPs (Figure S9A). By excluding these SNPs from the variant sites identified by SPRINT, the A>G% is further elevated (from 96.1% to 98.4%) but the number of A>G sites only slightly decreased from 121,888 to 121,621 (Figure S9B). This suggests the reliability of the RNA editing sites.

Editing level was defined as alternative reads count (G) divided by the total depth of an editing site. Variations were annotated with SnpEff v4.5.^93^ Editing sites were classified into different categories regarding their genomic mutation. For species without genomic annotation of UTR (untranslated region), there was not a UTR category of editing sites either.

The “bi-directional” editing sites referred to the sites located in a genomic region that both strands were able to transcribe. Since the RNA-Seq data were non-strand-specific, an A-to-G variation identified in the positive strand gene might actually come from a T-to-C variation in the negative strand transcripts, and vice versa. These variations were not A-to-I RNA editing sites. However, there was no way to distinguish the strand information from the data, so all variations in the “bi-directional” regions were removed.

To further show the reliability of the RNA editing detection scheme, we searched and found highly conserved RNA editing sites^100^ across distantly related species like *Drosophila* (Diptera) and Hemiptera (Figure S9C). This recoding site leads to a Tyr>Cys change in the protein sequence of potassium channel Shab. Since this Tyr>Cys RNA editing site in *Drosophila* and other hemipteran species like *Coridius chinensis* has been well acknowledged,^101^ the conserved editing events found in our three hemipteran species is likely to be true. Finally, since the DNA polymorphisms have been called, then in the RNA-Seq data we were able to test the linkage disequilibrium (LD) between SNP sites or between RNA editing sites. In theory, as our previous study revealed, RNA editing sites should have much weaker LD than genomic SNPs in RNA-Seq data.^25^ To our expectation, the LD between SNPs is very strong even in the RNA-Seq reads, while RNA editing exhibits much lower LD (Figure S9D). This pattern suggests that SNPs did not seem to largely infiltrate in the identified A-to-I RNA editing sites.

Meanwhile, we also acknowledged that any indirect inference or validation is no better than a perfect design of a transcriptome with a matched DNA-resequencing from the same individual(s), and this is the limitation of our current procedures.

### Quantification and statistical analysis

Bioinformatic analysis was described in the method details section. Gene expression of each ortho-group was measured by RPKM (reads per kilobase per million mapped reads). In the boxplot of RPKM each element represents one OG and n = the number of OGs. *p* values were calculated with Wilcoxon rank sum tests. In the comparison of exon or transcript numbers, we counted the number of distinct transcript IDs and exons per gene in each OG. Each element represents one OG and n = the number of OGs. *p* values were calculated using Wilcoxon rank sum tests. In the analysis of A-to-I RNA editing sites, the fraction of edited genes of each OG was determined. *p* values were assessed using Fisher’s exact test. In the analysis of gene numbers of each OG, the mean (±S.E.M.) of gene numbers in predaceous versus phytophagous species for each OG were shown as barplots. *p* values were calculated by T-test on the gene numbers with log_2_ (n+1) transformation.

## Supplemental information

Document S1. Figures S1–S9, Tables S1–S5, S7, and S8

Table S6. Ortho-groups identified across R. fuscipes, A. lucorum, and R. pedestris (EXCEL), related to Figure 2 The 1, 2, 3 in column names refer to the three species in order.

## References

[1] Johnsona K.P., Dietrich C.H., Friedrich F., Beutel R.G., Wipfler B., Peters R.S., Allena J.M., Petersen M., Donath A., Walden K.K.O. (2018). Phylogenomics and the evolution of hemipteroid insects. Proc. Natl. Acad. Sci. USA.

[2] McKennaa D.D., Shina S., Ahrens i., Balke M., Beza-Beza C., Clarkea D.J., Donathe A., Escalonae H.E., Friedrich F., Letsch H. (2019). The evolution and genomic basis of beetle diversity. Proc. Natl. Acad. Sci. USA.

[3] Misof B., Liu S., Meusemann K., Peters R.S., Donath A., Mayer C., Frandsen P.B., Ware J., Flouri T., Beutel R.G. (2014). Phylogenomics resolves the timing and pattern of insect evolution. Science.

[4] Polanczyk R.A., Pratissoli D. (2009). Biological control of agricultural pests: principles and field applications. Rev. Ceres.

[5] van Lenteren J.C. (2012). The state of commercial augmentative biological control: plenty of natural enemies, but a frustrating lack of uptake. BioControl.

[6] Li B., Duan Y., Du Z., Wang X., Liu S., Feng Z., Tian L., Song F., Yang H., Cai W. (2024). Natural selection and genetic diversity maintenance in a parasitic wasp during continuous biological control application. Nat. Commun..

[7] Zha S., Wang Z., Li X., Chen Z., Wang J., Li H., Cai W., Tian L. (2023). Microstructural adaptation for prey manipulation in the millipede assassin bugs (Hemiptera: Reduviidae: Ectrichodiinae). Biology.

[8] Gordon E.R., Weirauch C. (2016). Efficient capture of natural history data reveals prey conservatism of cryptic termite predators. Mol. Phylogenet. Evol..

[9] Messelink G.J., Janssen A. (2014). Increased control of thrips and aphids in greenhouses with two species of generalist predatory bugs involved in intraguild predation. Biol. Control.

[10] Henry T.J. (2017). Insect Biodiversity: Science and Society.

[11] Weirauch C., Bérenger J., Berniker L., Forero D., Forthman M., Frankenberg S., Freedman A., Gordon E., Hoey-Chamberlain R., Hwang W. (2014). An illustrated identification key to assassin bug subfamilies and tribes (Hemiptera: Reduviidae). Can. J. Arthropod Identif..

[12] Biswas B., Mitra B. (2011). Checklist of Indian assassin bugs (Insecta: Hemiptera: Reduviidae). Zool. Surv. India..

[13] Service M.W. (1980). A Guide to Medical Entomology.

[14] Koss A., Snyder W. (2005). Alternative prey disrupt biocontrol by a guild of generalist predators. Biol. Control.

[15] Forthman M., Weirauch C. (2012). Toxic associations: a review of the predatory behaviors of millipede assassin bugs (Hemiptera: Reduviidae: Ectrichodiinae). Eur. J. Entomol..

[16] Sahayaraj K. (2003). Hunter reduviids in cotton bug control. Acrobiose.

[17] Tomson M., Sahayaraj K., Kumar V., Avery P.B., McKenzie C.L., Osborne L.S. (2017). Mass rearing and augmentative biological control evaluation of *Rhynocoris fuscipes* (Hemiptera: Reduviidae) against multiple pests of cotton. Pest Manag. Sci..

[18] Kalidas S., Sahayaraj K. (2012). Survey of reduviids in cotton agro-ecosystem of Tamil Nadu, India. Middle East J. Sci. Res..

[19] Ambrose D., Claver M. (1997). Functional and numerical responses of the reduviid predator, *Rhynocoris fuscipes* F. (Het., Reduviidae) to cotton leafworm *Spodoptera litura* F. (Lep., Noctuidae). J. Appl. Entomol..

[20] Claver M.A., Ambrose D.P. (2002). Functional response of the predator, *Rhynocoris fuscipes* (Heteroptera: Reduviidae) to three pests of pigeon pea. Shashpa.

[21] Chakravarty S., Agnihotri M., Jagdish J. (2017). Seasonal abundance of predatory bugs, *Eocanthecona furcellata* and *Rhynocoris fuscipes* and its olfactory responses towards plant and pest mediated semiochemical cues in pigeonpea ecosystem. Legume Res..

[22] Crick F. (1970). Central dogma of molecular biology. Nature.

[23] Zhang P., Zhu Y., Guo Q., Li J., Zhan X., Yu H., Xie N., Tan H., Lundholm N., Garcia-Cuetos L. (2023). On the origin and evolution of RNA editing in metazoans. Cell Rep..

[24] Duan Y., Li H., Cai W. (2023). Adaptation of A-to-I RNA editing in bacteria, fungi, and animals. Front. Microbiol..

[25] Duan Y., Xu Y., Song F., Tian L., Cai W., Li H. (2023). Differential adaptive RNA editing signals between insects and plants revealed by a new measurement termed haplotype diversity. Biol. Direct.

[26] Eisenberg E., Levanon E.Y. (2018). A-to-I RNA editing - immune protector and transcriptome diversifier. Nat. Rev. Genet..

[27] Duan Y., Cai W., Li H. (2023). Chloroplast C-to-U RNA editing in vascular plants is adaptive due to its restorative effect: testing the restorative hypothesis. RNA.

[28] Duan Y., Ma L., Song F., Tian L., Cai W., Li H. (2023). Autorecoding A-to-I RNA editing sites in the *Adar* gene underwent compensatory gains and losses in major insect clades. RNA.

[29] Ma L., Zheng C., Xu S., Xu Y., Song F., Tian L., Cai W., Li H., Duan Y. (2023). A full repertoire of Hemiptera genomes reveals a multi-step evolutionary trajectory of auto-RNA editing site in insect *Adar* gene. RNA Biol..

[30] Zhao T., Ma L., Xu S., Cai W., Li H., Duan Y. (2024). Narrowing down the candidates of beneficial A-to-I RNA editing by comparing the recoding sites with uneditable counterparts. Nucleus (Calcutta).

[31] Gommans W.M., Mullen S.P., Maas S. (2009). RNA editing: a driving force for adaptive evolution?. Bioessays.

[32] Graveley B.R., Brooks A.N., Carlson J.W., Duff M.O., Landolin J.M., Yang L., Artieri C.G., van Baren M.J., Boley N., Booth B.W. (2011). The developmental transcriptome of *Drosophila melanogaster*. Nature.

[33] Wright C.J., Smith C.W.J., Jiggins C.D. (2022). Alternative splicing as a source of phenotypic diversity. Nat. Rev. Genet..

[34] Liu Y., Liu H., Wang H., Huang T., Liu B., Yang B., Yin L., Li B., Zhang Y., Zhang S. (2021). *Apolygus lucorum* genome provides insights into omnivorousness and mesophyll feeding. Mol. Ecol. Resour..

[35] Huang H.J., Ye Y.X., Ye Z.X., Yan X.T., Wang X., Wei Z.Y., Chen J.P., Li J.M., Sun Z.T., Zhang C.X. (2021). Chromosome-level genome assembly of the bean bug *Riptortus pedestris*. Mol. Ecol. Resour..

[36] Shoshan Y., Liscovitch-Brauer N., Rosenthal J.J.C., Eisenberg E. (2021). Adaptive proteome diversification by nonsynonymous A-to-I RNA editing in coleoid cephalopods. Mol. Biol. Evol..

[37] Zhan D., Zheng C., Cai W., Li H., Duan Y. (2023). The many roles of A-to-I RNA editing in animals: functional or adaptive?. Front. Biosci..

[38] Zhang Y., Duan Y. (2023). Genome-wide analysis on driver and passenger RNA editing sites suggests an underestimation of adaptive signals in insects. Genes.

[39] Porath H.T., Hazan E., Shpigler H., Cohen M., Band M., Ben-Shahar Y., Levanon E.Y., Eisenberg E., Bloch G. (2019). RNA editing is abundant and correlates with task performance in a social bumblebee. Nat. Commun..

[40] Porath H.T., Knisbacher B.A., Eisenberg E., Levanon E.Y. (2017). Massive A-to-I RNA editing is common across the metazoa and correlates with dsRNA abundance. Genome Biol..

[41] Hsiao Y.H.E., Bahn J.H., Yang Y., Lin X.Z., Tran S., Yang E.W., Quinones-Valdez G., Xiao X.S. (2018). RNA editing in nascent RNA affects pre-mRNA splicing. Genome Res..

[42] Mazloomian A., Meyer I.M. (2015). Genome-wide identification and characterization of tissue-specific RNA editing events in *D. melanogaster* and their potential role in regulating alternative splicing. RNA Biol..

[43] Rueter S.M., Dawson T.R., Emeson R.B. (1999). Regulation of alternative splicing by RNA editing. Nature.

[44] Liddicoat B.J., Piskol R., Chalk A.M., Ramaswami G., Higuchi M., Hartner J.C., Li J.B., Seeburg P.H., Walkley C.R. (2015). RNA editing by *ADAR1* prevents *MDA5* sensing of endogenous dsRNA as nonself. Science.

[45] Liang L., Cao C., Ji L., Cai Z., Wang D., Ye R., Chen J., Yu X., Zhou J., Bai Z. (2023). Complementary *Alu* sequences mediate enhancer-promoter selectivity. Nature.

[46] Tan M.H., Li Q., Shanmugam R., Piskol R., Kohler J., Young A.N., Liu K.I., Zhang R., Ramaswami G., Ariyoshi K. (2017). Dynamic landscape and regulation of RNA editing in mammals. Nature.

[47] Hedstrom L. (2002). Serine protease mechanism and specificity. Chem. Rev..

[48] Krem M.M., Di Cera E. (2001). Molecular markers of serine protease evolution. EMBO J..

[49] Di Cera E. (2009). Serine proteases. IUBMB Life.

[50] Nebert D.W., Russell D.W. (2002). Clinical importance of the cytochromes P450. Lancet.

[51] Arnér E.S., Holmgren A. (2000). Physiological functions of thioredoxin and thioredoxin reductase. Eur. J. Biochem..

[52] Holmgren A. (1989). Thioredoxin and glutaredoxin systems. J. Biol. Chem..

[53] Dermauw W., Van Leeuwen T. (2014). The ABC gene family in arthropods: comparative genomics and role in insecticide transport and resistance. Insect Biochem. Mol. Biol..

[54] Heckel D.G. (2012). Learning the ABCs of Bt: ABC transporters and insect resistance to *Bacillus thuringiensis* provide clues to a crucial step in toxin mode of action. Pestic. Biochem. Physiol..

[55] Liscovitch-Brauer N., Alon S., Porath H.T., Elstein B., Unger R., Ziv T., Admon A., Levanon E.Y., Rosenthal J.J.C., Eisenberg E. (2017). Trade-off between transcriptome plasticity and genome evolution in cephalopods. Cell.

[56] Liu Z., Zhang J. (2018). Most m6A RNA modifications in protein-coding regions are evolutionarily unconserved and likely nonfunctional. Mol. Biol. Evol..

[57] Chen S., Zhou Y., Chen Y., Gu J. (2018). Fastp: an ultra-fast all-in-one fastq preprocessor. Bioinformatics.

[58] Marcais G., Kingsford C. (2011). A fast, lock-free approach for efficient parallel counting of occurrences of k-mers. Bioinformatics.

[59] Vurture G.W., Sedlazeck F.J., Nattestad M., Underwood C.J., Fang H., Gurtowski J., Schatz M.C. (2017). Genomescope: fast reference-free genome profiling from short reads. Bioinformatics.

[60] Nurk S., Walenz B.P., Rhie A., Vollger M.R., Koren S., Grothe R., Miga K.H., Eichler E.E., Phillippy A.M., Koren S. (2020). HiCanu: accurate assembly of segmental duplications, satellites, and allelic variants from high-fidelity long reads. Genome Res..

[61] Guan D., McCarthy S.A., Wood J., Howe K., Wang Y., Durbin R. (2020). Identifying and removing haplotypic duplication in primary genome assemblies. Bioinformatics.

[62] Li H., Durbin R. (2009). Fast and accurate short read alignment with Burrows-Wheeler transform. Bioinformatics.

[63] Durand N.C., Shamim M.S., Machol I., Rao S.S., Huntley M.H., Lander E.S., Aiden E.L. (2016). Juicer provides a one-click system for analyzing loop-resolution Hi-C experiments. Cell Syst..

[64] Dudchenko O., Batra S.S., Omer A.D., Nyquist S.K., Hoeger M., Durand N.C., Shamim M.S., Machol I., Lander E.S., Aiden A.P., Aiden E.L. (2017). De novo assembly of the *Aedes aegypti* genome using Hi-C yields chromosome-length scaffolds. Science.

[65] Simão F.A., Waterhouse R.M., loannidis P., Kriventseva E.V., Evgeny M Z. (2015). BUSCO: assessing genome assembly and annotation completeness with single-copy orthologs. Bioinformatics.

[66] Rhie A., Walenz B.P., Koren S., Phillippy A.M. (2020). Merqury: reference-free quality, completeness, and phasing assessment for genome assemblies. Genome Biol..

[67] Ou S., Jiang N. (2019). LTR_FINDER_parallel: parallelization of LTR_FINDER enabling rapid identification of long terminal repeat retrotransposons. Mob. DNA.

[68] Chen N. (2004). Using RepeatMasker to identify repetitive elements in genomic sequences. Curr. Protoc. Bioinformatics.

[69] Jurka J., Kapitonov V.V., Pavlicek A., Klonowski P., Kohany O., Walichiewicz J. (2005). Repbase update, a database of eukaryotic repetitive elements. Cytogenet. Genome Res..

[70] Benso G. (1999). Tandem repeats finder: a program to analyze DNA sequences. Nucleic Acids Res..

[71] Kim D., Langmead B., Salzberg S.L. (2015). HISAT: a fast spliced aligner with low memory requirements. Nat. Methods.

[72] Kovaka S., Zimin A.V., Pertea G.M., Razaghi R., Salzberg S.L., Pertea M. (2019). Transcriptome assembly from long-read RNA-seq alignments with StringTie2. Genome Biol..

[73] Slater G.S.C., Birney E. (2005). Automated generation of heuristics for biological sequence comparison.. BMC Bioinformatics..

[74] Stanke M., Keller O., Gunduz I., Hayes A., Waack S., Morgenstern B. (2006). AUGUSTUS: ab initio prediction of alternative transcripts. Nucleic Acids Res..

[75] Cantarel B.L., Korf I., Robb S.M., Parra G., Ross E., Moore B., Holt C., Alvarado A.S., Yandell M. (2008). MAKER: an easy-to-use annotation pipeline designed for emerging model organism genomes. Genome Res..

[76] Cantalapiedra C.P., Hernández-Plaza A., Letunic I., Bork P., Huerta-Cepas J. (2021). eggNOG-mapper v2: functional annotation, orthology assignments, and domain prediction at the metagenomic scale. Mol. Biol. Evol..

[77] Jones P., Binns D., Chang H.-Y., Fraser M., Li W., McAnulla C., McWilliam H., Maslen J., Mitchell A., Nuka G. (2014). InterProScan 5: genome-scale protein function classification. Bioinformatics.

[78] Camacho C., Coulouris G., Avagyan V., Ma N., Papadopoulos J., Bealer K., Madden T.L. (2009). BLAST+: architecture and applications. BMC Bioinformatics..

[79] Finn R.D., Clements J., Eddy S.R. (2011). HMMER web server: interactive sequence similarity searching. Nucleic Acids Res..

[80] Tang H., Bowers J.E., Wang X., Ming R., Alam M., Paterson A. (2008). Synteny and collinearity in plant genomes. Science.

[81] Emms D.M., Kelly S. (2019). OrthoFinder: phylogenetic orthology inference for comparative genomics. Genome Biol..

[82] Katoh K., Standley D.M. (2013). MAFFT multiple sequence alignment software version 7: improvements in performance and usability. Mol. Biol. Evol..

[83] Steenwyk J.L., Buida III T.J., Labella A.L., Li Y., Shen X.-X., Rokas A. (2021). PhyKIT: a broadly applicable UNIX shell toolkit for processing and analyzing phylogenomic data. Bioinformatics.

[84] Dobin A., Davis C.A., Schlesinger F., Drenkow J., Zaleski C., Jha S., Batut P., Chaisson M., Gingeras T.R. (2013). STAR: ultrafast universal RNA-seq aligner. Bioinformatics.

[85] Liao Y., Smyth G.K., Shi W. (2014). FeatureCounts: an efficient general purpose program for assigning sequence reads to genomic features. Bioinformatics.

[86] Capella-Gutiérrez S., Silla-Martínez J.M., Gabaldón T. (2009). TRIMAl: a tool for automated alignment trimming in large-scale phylogenetic analyses. Bioinformatics.

[87] Kück P., Longo G.C. (2014). FASconCAT-G: extensive functions for multiple sequence alignment preparations concerning phylogenetic studies. Front. Zool..

[88] Kück P., Struck T.H. (2014). BaCoCa--a heuristic software tool for the parallel assessment of sequence biases in hundreds of gene and taxon partitions. Mol. Phylogenet. Evol..

[89] Nguyen L.-T., Schmidt H.A., Von Haeseler A., Minh B.Q. (2015). IQ-TREE: a fast and effective stochastic algorithm for estimating maximum-likelihood phylogenies. Mol. Biol. Evol..

[90] Bie T.D., Cristianini N., Demuth J.P., Hahn M.W. (2006). CAFE: a computational tool for the study of gene family evolution. Bioinformatics.

[91] Li H. (2018). Minimap2: pairwise alignment for nucleotide sequences. Bioinformatics.

[92] Zhang F., Lu Y., Yan S., Xing Q., Tian W. (2017). SPRINT: an SNP-free toolkit for identifying RNA editing sites. Bioinformatics.

[93] Cingolani P., Platts A., Wang le L., Coon M., Nguyen T., Wang L., Land S.J., Lu X., Ruden D.M. (2012). A program for annotating and predicting the effects of single nucleotide polymorphisms, SnpEff: SNPs in the genome of *Drosophila melanogaster* strain w1118; iso-2; iso-3. Fly.

[94] Poggio M., Bressa M., Papeschi A. (2007). Karyotype evolution in Reduviidae (Insecta: Heteroptera) with special reference to Stenopodainae and Harpactorinae. Comp. Cytogenet..

[95] Liu Q., Guo Y., Zhang Y., Hu W., Li Y., Zhu D., Zhou Z., Wu J., Chen N., Zhou X.-N. (2019). A chromosomal-level genome assembly for the insect vector for Chagas disease, *Triatoma rubrofasciata*. GigaScience.

[96] Luca F., Perry G., Di Rienzo A. (2010). Evolutionary adaptations to dietary changes. Annu. Rev. Nutr..

[97] Bai Y., Shi Z., Zhou W., Wang G., Shi X., He K., Li F., Zhu Z.R. (2022). Chromosome-level genome assembly of the mirid predator *Cyrtorhinus lividipennis* Reuter (Hemiptera: Miridae), an important natural enemy in the rice ecosystem. Mol. Ecol. Resour..

[98] Walker A.A., Mayhew M.L., Jin J., Herzig V., Undheim E.A.B., Sombke A., Fry B.G., Meritt D.J., King G.F. (2018). The assassin bug *Pristhesancus plagipennis* produces two distinct venoms in separate gland lumens. Nat. Commun..

[99] Zheng C., Ma L., Song F., Tian L., Cai W., Li H., Duan Y. (2024). Comparative genomic analyses reveal evidence for adaptive A-to-I RNA editing in insect *Adar* gene. Epigenetics.

[100] Liu J., Zheng C., Duan Y. (2024). New comparative genomic evidence supporting the proteomic diversification role of A-to-I RNA editing in insects. Mol. Genet. Genom..

[101] Duan Y., Ma L., Liu J., Liu X., Song F., Tian L., Cai W., Li H. (2024). The first A-to-I RNA editome of hemipteran species *Coridius chinensis* reveals overrepresented recoding and prevalent intron editing in early-diverging insects. Cell. Mol. Life Sci..

