# Supplementary material for: Comparative genomic analyses on assassin bug Rhynocoris fuscipes (Hemiptera: Reduviidae) reveal genetic bases governing the diet-shift
Source: iScience. 2024 Jun 28;27(8):110411. doi: 10.1016/j.isci.2024.110411 (PMC11301091; doi:10.1016/j.isci.2024.110411)
Supplement: Document S1. Figures S1–S9, Tables S1–S5, S7, and S8 [file mmc1.pdf]

iScience, Volume *27*

Supplemental information

Comparative genomic analyses on assassin bug *Rhynocoris fuscipes* (Hemiptera: Reduviidae) reveal genetic bases governing the diet-shift

Ling Ma, Yuange Duan, Yunfei Wu, Hailin Yang, Haibin Deng, Xinzhi Liu, Tianyou Zhao, Yisheng Zhao, Li Tian, Fan Song, Teiji Sota, Wanzhi Cai, and Hu Li

Supplementary Figures

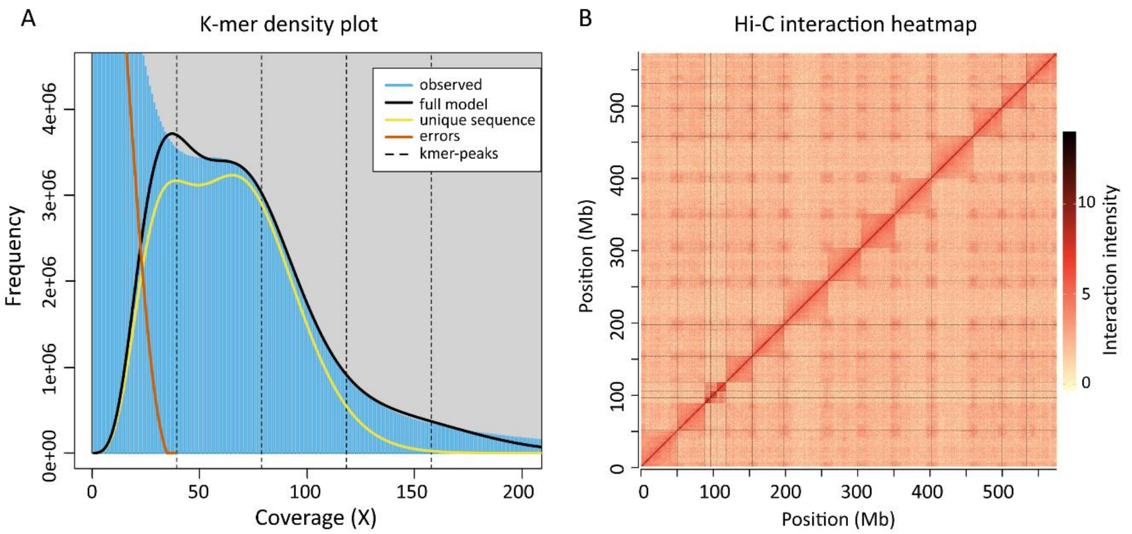

Figure S1. Supporting information of *R. fuscipes* genome assembly, related to Figure 1. (A) GenomeScope profile with 17-mer. The fit of the model (black line) to the observed k-mer density plot. (B) Genome-wide Hi-C link heatmap of the final chromosome. Each scaffold refers to chromosome and each pixel refers to a 100-Kb bin.

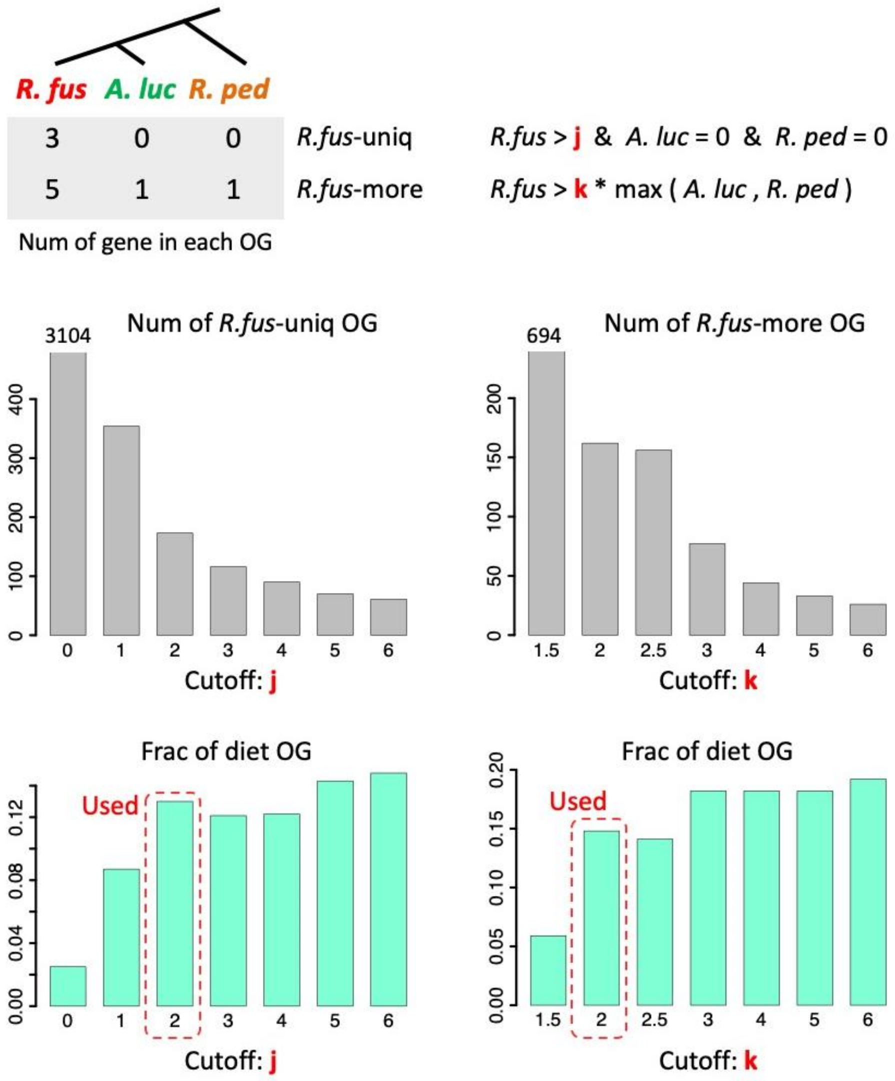

Figure S2. Numbers of *R. fus*-unique and *R. fus*-more OGs under different cutoffs, related to Figure 2 and Figure 5. Grey bars represent numbers of total OGs. Blue bars represent fraction of diet OGs. The definition of *R. fus*-unique OG is: *R. fus* > j & *A. luc* = 0 & *R. ped* = 0. Here, j = (0, 1, 2, 3, 4, 5, 6) were shown and j = 2 was used in the downstream analysis. The definition of *R. fus*-more OG is: *R. fus* > k*max (*A. luc*, *R. ped*). Here, k = (1.5, 2, 2.5, 3, 4, 5, 6) were shown and k = 2 was used in the downstream analysis.

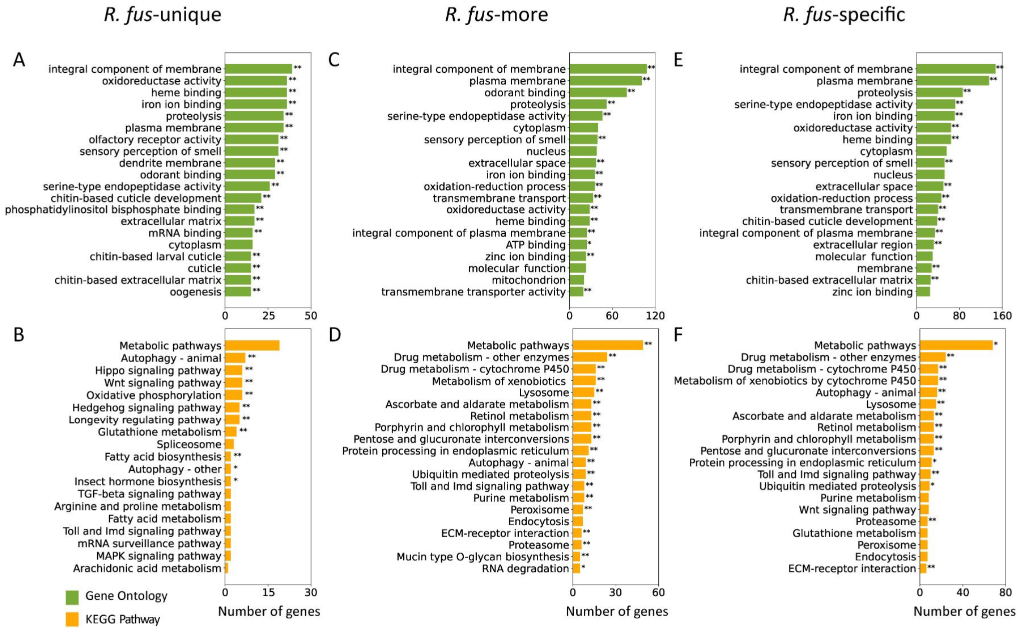

Figure S3. GO and KEGG analysis of diet genes in *R. fuscipes*, related to Figure 2. These plots show the top 20 enriched terms from Gene Ontology (green) or pathways from KEGG (yellow) for three categories: *R. fus*-unique (A-B), *R. fus*-more (C-D) and *R. fus*-specific (E-F). The X-axis represents the number of enriched genes and the Y-axis represents the enriched terms and pathways. Significant enrichment is marked with asterisks: *, $P < 0.05$; **, $P < 0.01$.

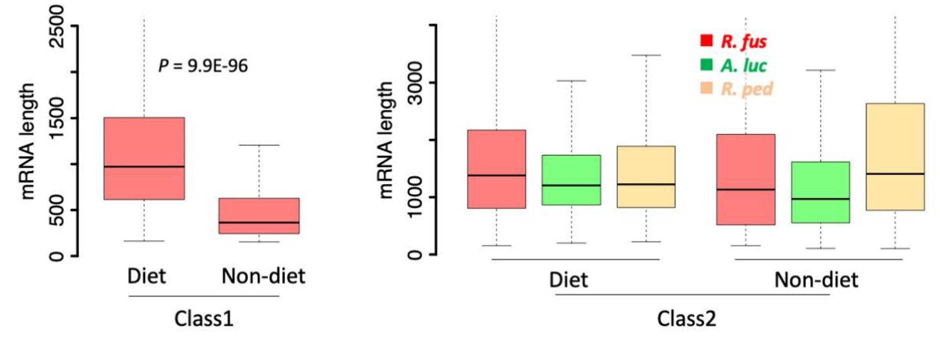

Figure S4. mRNA length of different categories of genes, related to Figure 2. Left panel: Class1 (*R. fus*-specific) genes. Right panel: Class2 (shared) genes. *P* value was calculated by Wilcoxon rank sum test.

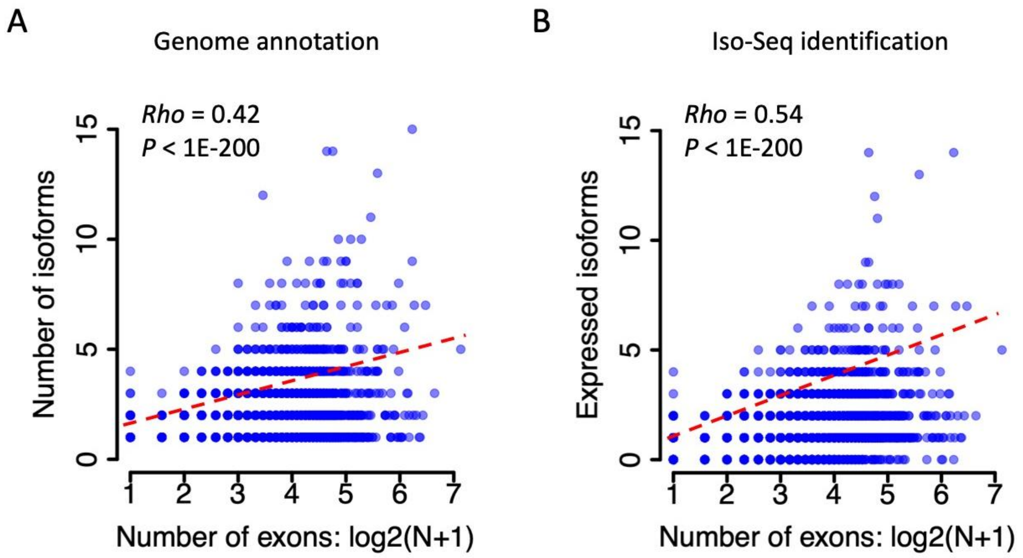

Figure S5. Genes with more exons tend to have more isoforms, related to Figure 2. (A) Spearman correlation between number of exons (X-axis) and number of transcript isoforms (Y-axis) annotated in the *R. fuscipes* reference genome. (B) From Iso-Seq results, the numbers of expressed transcript isoforms per gene increase with number of exons.

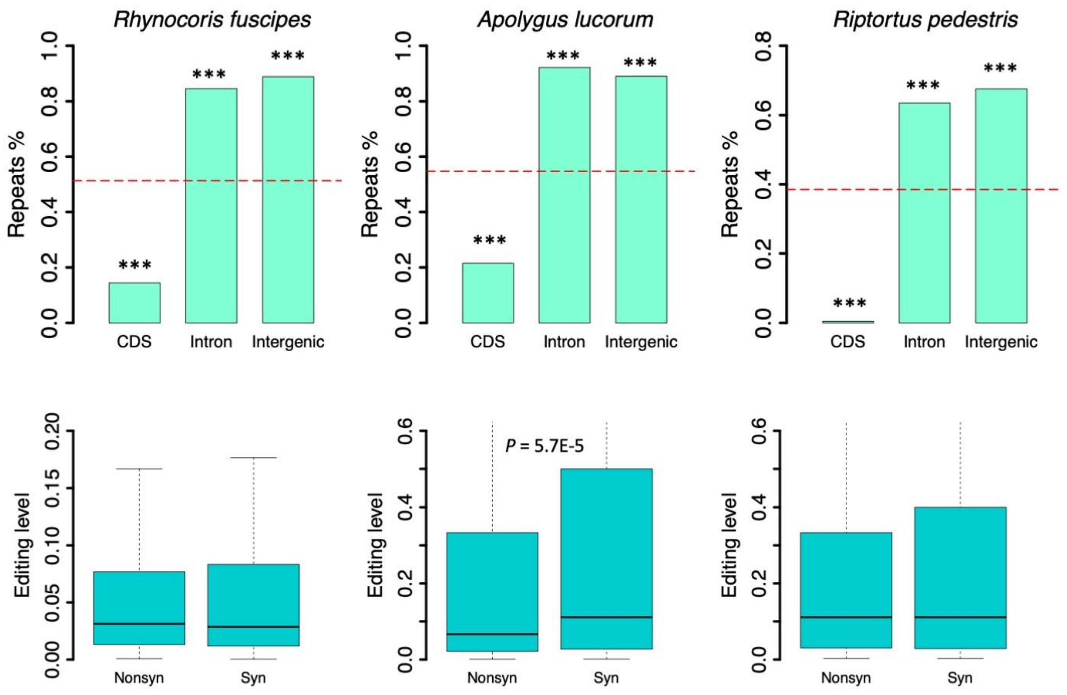

Figure S6. Distribution and editing levels of RNA editing sites in three species, related to Figure 3 and Figure 4. Upper panel: the fraction of editing sites in genomic repeat regions. Red dashed lines represent the baselines of genomic repeat content in each species. *P* values were calculated using Fisher's exact test comparing to the baseline. ***, $P < 0.001$. Lower panel: editing levels of nonsynonymous and synonymous sites. *P* value was calculated by Wilcoxon rank sum test.

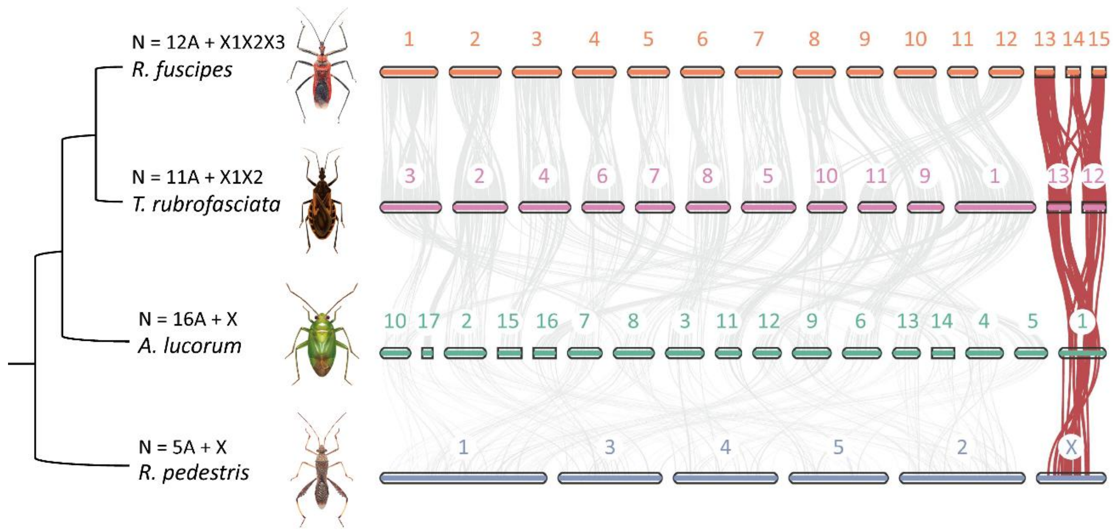

Figure S7. Synteny analysis of *R. fuscipes* and other hemipteran species, related to Figure 1 and the STAR Methods. Species were ordered by their phylogeny.

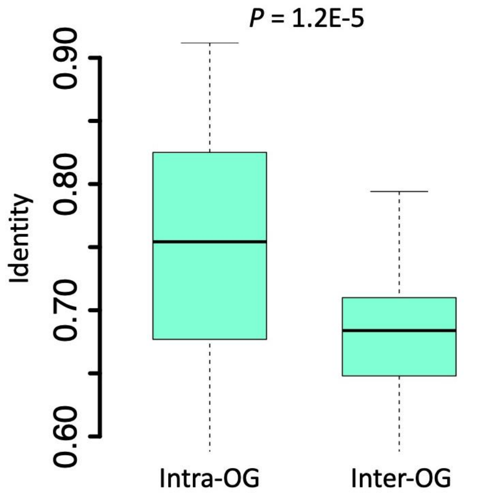

Figure S8. Comparison of intra-OG similarity and inter-OG similarity, related to the STAR Methods. *P* value was calculated by Wilcoxon rank sum test.

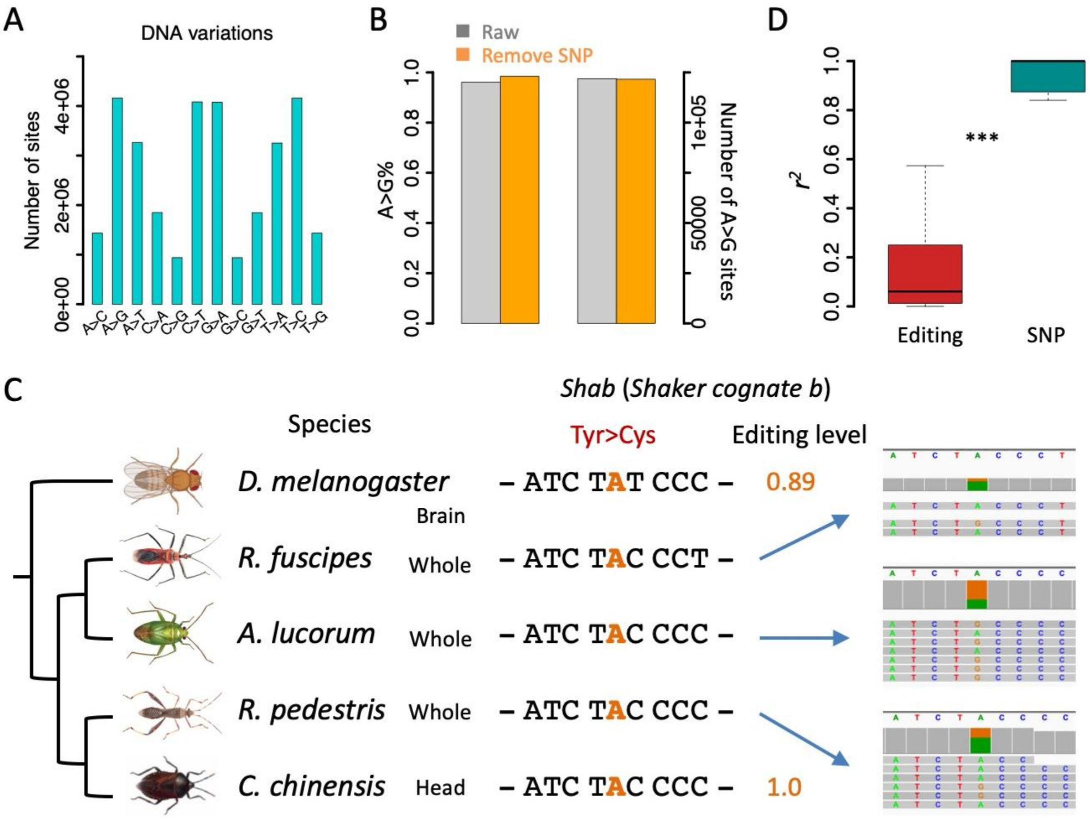

Figure S9. The proof or improvement of the reliability of A-to-I RNA editing sites in *R. fuscipes*, related to Figure 3 and the STAR Methods. (A) SNPs identified from the WGS data of *R. fuscipes*. (B) The fractions of A>G variations before and after the exclusion of potential SNPs. (C) A highly conserved RNA editing site in potassium channel gene *Shab* which is identified in *Drosophila melanogaster* (Diptera), *Coridius chinensis* (Hemiptera) and the three hemipteran species we used. (D) Linkage disequilibrium (LD) between SNPs or between RNA editing sites in the RNA-Seq data.

Supplementary Tables

Table S1. Statistics for sequencing data generated in this study, related to Figure 1.

Types of sequencing	Platform	Raw Data (bp)	Clean data (bp)	Coverage (×)
HiFi reads	PacBio Sequel II	389,081,852,845	26,193,345,706	46.49
DNA-seq data	Illumina	51,162,820,500	49,642,286,802	88.11
Hi-C data	Illumina	58,121,334,900	55,395,256,430	98.32
RNA-seq data	Illumina	7,335,070,800	6,875,756,672	12.20
Full-length transcriptome	PacBio Sequel II	17,853,889,221	458,948,578	31.69

Table S2. Genome estimation of *Rhynocoris fuscipes*, related to Figure 1.

K-mer	Genome size (bp)	Heterozygosity (%)
17	563,387,433	1.81

Table S3. Statistics of *Rhynocoris fuscipes* genome assembly at contig-level and chromosome-level, related to Figure 1 and Table 1.

Types	Contig-level genome	Chromosome-level genome
Longest length (bp)	34,075,473	63,527,133
N10 (bp)	15,687,846	63,527,133
N20 (bp)	11,262,173	61,338,067
N30 (bp)	8,536,424	51,431,823
N40 (bp)	5,440,018	48,777,691
N50 (bp)	3,929,296	47,563,414
N60 (bp)	2,602,328	43,916,315
N70 (bp)	1,860,152	40,735,142
N80 (bp)	1,041,268	37,434,878
N90 (np)	463,975	35,276,945
Total length (bp)	621,025,795	621,561,295
contig number	1,159	90
GC (%)	31.50	31.50

Table S4. Statistics of gene annotation of *Rhynocoris fuscipes*, related to Table 1.

Type	Value
Gene counts	17,486
Max gene length	146,766
Min gene length	153
Average gene length	7,210.26
mRNA counts	21,793
max mRNA length	24,792
min mRNA length	153
Average mRNA length	1,573.19
Average mRNA number	1
CDS counts	21,793
Max CDS length	24,441
Min CDS length	150
Average CDS length	1,218
Average protein length	406
Average exon length	227
Average exon counts	7

Table S5. Statistics of repeat content of *Rhynocoris fuscipes* and two other non-predaceous true bugs, related to Figure 1.

Species	Class	Subfamily	Masked length (bp)	Percent (%)
Rhynocoris fuscipes	LTR Retrotransposon	Copia	127,977	0.02
		Gypsy	10,043,005	1.62
		Unknown	51,560,331	8.30
	DNA Transposon	CACTA	26,049,434	4.19
		Mutator	91,248,025	14.68
		PIF/Harbinger	4,328,688	0.70
		Tc1/Mariner	14,467,706	2.33
		hAT	97,663,138	15.71
		Helitron	23,147,487	3.72
Apolygus lucorum	LTR Retrotransposon	Copia	17,041,485	1.66
		Gypsy	119,654,790	11.69
		Unknown	100,888,597	9.86
	DNA Transposon	CACTA	37,147,402	3.63
		Mutator	101,507,236	9.92
		PIF/Harbinger	16,649,385	1.63
		Tc1/Mariner	6,796,505	0.66
		hAT	103,065,758	10.07
		Helitron	57,156,062	5.58
Riptortus pedestris	LTR Retrotransposon	Copia	5,823,959	0.54
		Gypsy	83,410,976	7.73
		Unknown	318,614,358	29.51
	DNA Transposon	CACTA	714,423	0.07
		Mutator	7,114,000	0.66
		PIF/Harbinger	156,317	0.01
		Tc1/Mariner	148,192	0.01
		hAT	1,216,708	0.11
		Helitron	807,426	0.07

Table S7. Information of protein domains collected from Pfam database, related to Figure 2.

Class	Gene family	Pfam motif
Detoxification-related	Cytochrome P450	PF00067
	Glutathione S-transferase	PF00043, PF02798, PF01124
	Choline/carboxylesterases	PF00135
	ATP-binding cassette transporter	PF00005
Chemosensory-related	Ionotropic receptors (IR)	PF00060
	Odorant receptors (OR)	PF13853, PF02949
	Gustatory receptors (GR)	PF06151, PF08395
	Odorant-binding proteins (OBP)	PF01395
	Chemosensory proteins (CSP)	PF03392
	Sensory neuron membrane proteins (SNMPs)	PF01130
Digestion-related	Serine protease (S1)	PF00089
	Serpin	PF00079
	Carboxypeptidase (M14)	PF00246
	Aspartic Protease (A1)	PF00026
	Lipase	PF00151, PF01764,, PF06350, PF04083, PF01734, PF00657, PF13472
	Alpha amylase	PF00128
	Cathepsin	PF08246, PF08773
	Thioredoxin	PF00085
	CUB	PF00431
	Ptu family	PF08117

Note: The Pfam database is a large collection of protein families, each represented by multiple sequence alignments and hidden Markov models (HMMs).

Table S8. Information of additional 32 species in Paraneoptera, related to Figure 6 and Figure 7.

Order	Suborder	Family	Species	Download site
Hemiptera	Heteroptera	Gerridae	*Gerris buenoi*	https://i5k.nal.usda.gov/Gerris_buenoi
		Anthocoridae	*Orius insidiosus*	http://v2.insect-genome.com/Organism/592
			Orius laevigatus	http://v2.insect-genome.com/Organism/593
		Miridae	*Cyrtorhinus lividipennis*	insect-genome.com/Cliv/
		Alydidae	*Riptortus pedestris*	http://v2.insect-genome.com/Organism/689
		Lygaeidae	*Oncopeltus fasciatus*	https://i5k.nal.usda.gov/Oncopeltus_fasciatus
		Pentatomidae	*Halyomorpha halys*	https://ftp.ncbi.nlm.nih.gov/genomes/all/GCF/000/696/795/GCF_000696795.2_Hhal_2.0
	Auchenorrhyncha	Delphacidae	*Laodelphax striatellus*	http://v2.insect-genome.com/Organism/477
			Sogatella furcifera	http://v2.insect-genome.com/Organism/709
			Nilaparvata lugens	https://ftp.ncbi.nlm.nih.gov/genomes/all/GCF/014/356/525/GCF_014356525.2_ASM1435652v1/
		Cicadellidae	*Homalodisca vitripennis*	https://ftp.ncbi.nlm.nih.gov/genomes/all/GCF/021/130/785/GCF_021130785.1_UT_GWSS_2.1/
	Sternorrhyncha	Aphididae	*Myzus persicae*	https://ftp.ncbi.nlm.nih.gov/genomes/all/GCF/001/856/785
			Aulacorthum solani	http://v2.insect-genome.com/Organism/100
			Diuraphis noxia	http://v2.insect-genome.com/Organism/244
			Sitobion miscanthi	http://v2.insect-genome.com/Organism/706
			Acyrthosiphon pisum	https://ftp.ncbi.nlm.nih.gov/genomes/all/GCF/005/508/785/GCF_005508785.2_pea_aphid_22Mar2018_4r6ur_v2
			Aphis craccivora	http://v2.insect-genome.com/Organism/76

			Aphis gossypii	https://ftp.ncbi.nlm.nih.gov/genomes/all/GCF/020/184/175
			Rhopalosiphum maidis	https://ftp.ncbi.nlm.nih.gov/genomes/all/GCF/003/676/215/GCF_003676215.2_ASM367621v3/
			Melanaphis sacchari	https://ftp.ncbi.nlm.nih.gov/genomes/all/GCF/002/803/265
			Sipha flava	https://ftp.ncbi.nlm.nih.gov/genomes/all/GCF/003/268/045
			Cinara cedri	http://v2.insect-genome.com/Organism/185
			Eriosoma lanigerum	http://v2.insect-genome.com/Organism/369
			Hormaphis cornu	http://v2.insect-genome.com/Organism/452
		Phylloxeridae	*Daktulosphaira vitifoliae*	http://v2.insect-genome.com/Organism/227
		Pseudococcidae	*Ferrisia virgata*	http://v2.insect-genome.com/Organism/391
			Trionymus perrisii	http://v2.insect-genome.com/Organism/776
			Phenacoccus solenopsis	http://v2.insect-genome.com/Organism/624
		Liviidae	*Diaphorina citri*	https://ftp.ncbi.nlm.nih.gov/genomes/all/GCF/000/475/195/GCF_000475195.1_Diaci_psyllid_genome_assembly_version_1.1/
		Aleyrodidae	*Trialeurodes vaporariorum*	http://v2.insect-genome.com/Organism/765
			Bemisia tabaci	https://ftp.ncbi.nlm.nih.gov/genomes/all/GCF/001/854/935/GCF_001854935.1_ASM185493v1
Thysanoptera	Terebrantia	Thripidae	*Thrips palmi*	https://ftp.ncbi.nlm.nih.gov/genomes/all/GCF/012/932/325
